# The therapeutic mechanism of transcranial iTBS on nerve regeneration and functional recovery in rats with complete spinal cord transection

**DOI:** 10.3389/fimmu.2023.1153516

**Published:** 2023-06-14

**Authors:** Jia-Lin Liu, Shuai Wang, Zheng-Hong Chen, Rong-Jie Wu, Hai-Yang Yu, Shang-Bin Yang, Jing Xu, Yi-Nan Guo, Ying Ding, Ge Li, Xiang Zeng, Yuan-Huan Ma, Yu-Lai Gong, Chuang-Ran Wu, Li-Xin Zhang, Yuan-Shan Zeng, Bi-Qin Lai

**Affiliations:** ^1^ Key Laboratory for Stem Cells and Tissue Engineering, Ministry of Education, Sun Yat-sen University, Guangzhou, Guangdong, China; ^2^ Rehabilitation Center, Shengjing Hospital Affiliated to China Medical University, Shenyang, Liaoning, China; ^3^ Rehabilitation Medicine Department, The First Affiliated Hospital of Sun Yat-sen University, Guangzhou, Guangdong, China; ^4^ Shantou University Medical College, Shantou, China; ^5^ Department of Orthopedics, Guangdong Provincial People’s Hospital, Guangdong Academy of Medical Sciences, Guangzhou, China; ^6^ Department of Histology and Embryology, Zhongshan School of Medicine, Sun Yat-sen University, Guangzhou, Guangdong, China; ^7^ Guangdong Provincial Key Laboratory of Brain Function and Disease, Zhongshan School of Medicine, Sun Yat-sen University, Guangzhou, China; ^8^ Institute of Spinal Cord Injury, Sun Yat-sen University, Guangzhou, China; ^9^ Medical Research Center, Guangdong Cardiovascular Institute, Guangdong Provincial People’s Hospital, Guangdong Academy of Medical Science, Guangzhou, China; ^10^ Guangzhou Institute of Clinical Medicine, Guangzhou First People’s Hospital, South China University of Technology, Guangzhou, China; ^11^ Department of Neurology, Sichuan Provincial Rehabilitation Hospital, Chengdu, China; ^12^ Co-innovation Center of Neuroregeneration, Nantong University, Nantong, China

**Keywords:** spinal cord injury, intermittent theta-burst stimulation (iTBS), nerve regeneration, synaptic plasticity, motor pathway, neuroprotection

## Abstract

**Background:**

After spinal cord transection injury, the inflammatory microenvironment formed at the injury site, and the cascade of effects generated by secondary injury, results in limited regeneration of injured axons and the apoptosis of neurons in the sensorimotor cortex (SMC). It is crucial to reverse these adverse processes for the recovery of voluntary movement. The mechanism of transcranial intermittent theta-burst stimulation (iTBS) as a new non-invasive neural regulation paradigm in promoting axonal regeneration and motor function repair was explored by means of a severe spinal cord transection.

**Methods:**

Rats underwent spinal cord transection and 2 mm resection of spinal cord at T10 level. Four groups were studied: Normal (no lesion), Control (lesion with no treatment), sham iTBS (lesion and no functional treatment) and experimental, exposed to transcranial iTBS, 72 h after spinal lesion. Each rat received treatment once a day for 5 days a week; behavioral tests were administered one a week. Inflammation, neuronal apoptosis, neuroprotective effects, regeneration and synaptic plasticity after spinal cord injury (SCI) were determined by immunofluorescence staining, western blotting and mRNA sequencing. For each rat, anterograde tracings were acquired from the SMC or the long descending propriospinal neurons and tested for cortical motor evoked potentials (CMEPs). Regeneration of the corticospinal tract (CST) and 5-hydroxytryptamine (5-HT) nerve fibers were analyzed 10 weeks after SCI.

**Results:**

When compared to the Control group, the iTBS group showed a reduced inflammatory response and reduced levels of neuronal apoptosis in the SMC when tested 2 weeks after treatment. Four weeks after SCI, the neuroimmune microenvironment at the injury site had improved in the iTBS group, and neuroprotective effects were evident, including the promotion of axonal regeneration and synaptic plasticity. After 8 weeks of iTBS treatment, there was a significant increase in CST regeneration in the region rostral to the site of injury. Furthermore, there was a significant increase in the number of 5-HT nerve fibers at the center of the injury site and the long descending propriospinal tract (LDPT) fibers in the region caudal to the site of injury. Moreover, CMEPs and hindlimb motor function were significantly improved.

**Conclusion:**

Neuronal activation and neural tracing further verified that iTBS had the potential to provide neuroprotective effects during the early stages of SCI and induce regeneration effects related to the descending motor pathways (CST, 5-HT and LDPT). Furthermore, our results revealed key relationships between neural pathway activation, neuroimmune regulation, neuroprotection and axonal regeneration, as well as the interaction network of key genes.

## 1 Introduction

Spinal cord injury (SCI) often leads to the severe impairment of sensory and voluntary motor function. The acute and chronic stages of SCI involve a series of destructive events, including ischemia, oxidative stress, inflammation, neuronal apoptosis and scar formation (1–4). A wide array of studies has confirmed that SCI can cause inflammation in the brain and the apoptosis of neurons in the sensorimotor cortex (SMC), thus exerting significant effect on the regeneration of brain-derived nerve fibers innervating the injury site (5–8). It has yet to be determined whether brain-derived nerve regeneration and motor function can be promoted by activating brain motor neural pathways after SCI as an insufficient body of research has addressed the underlying therapeutic strategies and mechanisms.

As a non-invasive, safe, and easily accepted form of treatment, transcranial magnetic stimulation (TMS) can exert a significant effect on neural networks in the brain by altering the excitability of neurons (9–13). Despite a body of clinical data supporting the efficacy of TMS for the treatment of SCI (14, 15), the specific cellular and molecular mechanisms targeted by TMS are still unclear. The stimulation paradigms of TMS used in clinical therapy include traditional repetitive TMS and theta-burst stimulation (TBS) (14, 16, 17). TBS is a novel TMS protocol that rapidly induces synaptic plasticity (17, 18). During TBS, short bursts of high frequency (50 Hz) stimulation are repeated at 5 Hz (200-ms interval) (19). TBS can be either intermittent (iTBS), with excitatory effects, or continuous, with inhibitory effects; these events are associated with long-term potentiation- and long-term depression-like activity, respectively. Over recent years, iTBS has been used increasingly as a treatment option to promote the recovery of neurological function due to its short treatment time, simple operative protocol, and clinical acceptability (17, 18, 20).

Over recent years, research on iTBS has mostly focused on the observation of therapeutic phenomena; there is a lack of in-depth and systematic research on the specific mechanisms involved (16–18). In a previous study, Marufa et al. (21) explored neuronal plasticity and the recovery of motor function following iTBS therapy in rats with spinal cord clipping injuries with different compression forces. These authors observed enhancement in the amplitude of cortical motor-evoked potentials (CMEPs) and an increase in the expression levels of growth-associated protein 43 (GAP43) in the brain and spinal cord. However, these authors did not observe a significant improvement of motor function in the hindlimbs. This may be related to the fact that Marufa et al. first applied iTBS therapy four weeks after SCI an used a short treatment period lasting two weeks. In another study, Delarue et al. (22) found that repetitive trans-spinal magnetic stimulation could improve neuroinflammation and regulate neuroglial scar formation by regulating microglia and astrocytes, thus promoting the repair of SCI. Therefore, we hypothesized that transcranial iTBS may also play a similar role in the regulation of neuroinflammation and neuroprotection. The key to recovering voluntary motor function is activating and maintaining neuroprotective effects on vital motor pathways.

The mechanisms underlying the effects of transcranial iTBS on axonal regeneration have rarely been studied in an animal model of complete spinal cord transection. The stimulation site was selected on the SMC, as this strategy could effectively explain the mechanisms underlying the effects of transcranial iTBS on promoting brain-derived axon regeneration and the repair of motor pathways. Previous treatments for SCI focused on axonal regeneration within specific lesions (23–25). However, in this study, we focused on the mechanisms of transcranial pathways on brain-derived axonal regeneration and investigated neuroprotective mechanisms by analyzing cells, proteins, and gene interactions in the acute and subacute phases of SCI and axonal regeneration and the repair of voluntary motor function in later phases by means of TMS of the SMC in a severe spinal cord transection rat model.

## 2 Material and methods

### 2.1 Experimental animals

Eight-week-old Sprague-Dawley rats (180–220 g) were provided by the Guangdong Medical Laboratory Animal Center. All animal protocols and animal handling procedures were approved by the ethics committee of Sun Yat-sen University (Animal Use Protocol No. 2021PS704K).

Animals were chosen from multiple cages and assigned randomly to each experiment. All rats were randomly divided into a normal group (*n* = 10), a Control group (*n* = 15), a sham iTBS group (*n* = 15), and an iTBS group (*n* = 15). Rats in the Normal group did not receive surgery or treatment. All animals in the Control group, the sham iTBS group and the iTBS group, received T10 spinal cord transection surgery. Rats in the Control group only received T10 spinal cord transection and did not receive iTBS treatment. Rats in the iTBS group were treated 72 hours after SCI for 8 weeks. Rats in the sham iTBS group were given sham stimulation with the same parameters 72 hours after SCI. Five rats (each) in the Normal group were sacrificed after 2 weeks and 4 weeks. Five rats (each) from other three groups were sacrificed 2 weeks, 4 weeks, and 10 weeks after SCI. Detailed animal groupings and handling procedures are given in **Table 1**. In addition, another six normal rats were randomly selected before spinal cord transection. Three rats were treated with iTBS for 5 days, and three rats were used as control without any measures to detect the expression of c-Fos in Map2^+^ neurons at SMC, C4 and T10 level of spinal cord.

**Table 1:** Animal groupings and handling procedures.

Group Handling	iTBS group	sham iTBS group	Control group	Normal group
**Before SCI**	MT detection	MT detection	MT detection	MT detection
**0h**	T10 spinal cord transection	T10 spinal cord transection	T10 spinal cord transection	No operation
**72h**	iTBS treatment (once a day, five days a week)	sham stimulation (once a day, five days a week)	No treatment	No treatment
**1w**	Behavioral testing (*n* = 10, every week)	Behavioral testing (*n* = 10, every week)	Behavioral testing (*n* = 10, every week)	Behavioral testing (*n* = 10, every week)
**2w**	Perfusion (*n* = 5) **IF:** SMC *(IBA-1, Cle-caspase3/NeuN)*; **WB:** SMC *(CD206/CD68/CCR7, Pro-caspase3/Cle-caspase3)*	Perfusion (*n* = 5) **IF:** SMC *(IBA-1, Cle-caspase3/NeuN)*; **WB:** SMC *(CD206/CD68/CCR7, Pro-caspase3/Cle-caspase3)*	Perfusion (*n* = 5) **IF:** SMC *(IBA-1, Cle-caspase3/NeuN)*; **WB:** SMC *(CD206/CD68/CCR7, Pro-caspase3/Cle-caspase3)*	Perfusion (*n* = 5) **IF:** SMC *(IBA-1, Cle-caspase3/NeuN)*; **WB:** SMC *(CD206/CD68/CCR7, Pro-caspase3/Cle-caspase3)*
**4w**	Perfusion (*n* = 5) **mRNA sequencing:** SMC/T10 SC; **IF:** T10 SC *(CD68, Cle-caspase3/NeuN, ATF3, GFAP, GAP43, NF)*; **WB:** T10 SC *(Pro-caspase3/Cle-caspase3,PSD95,SYN,NF,GAP43)*; **ELISA:** T10 SC *(BDNF)*	Perfusion (*n* = 5) **mRNA sequencing:** SMC/T10 SC; **IF:** T10 SC *(CD68, Cle-caspase3/NeuN, ATF3, GFAP, GAP43, NF)*; **WB:** T10 SC *(Pro-caspase3/Cle-caspase3,PSD95,SYN,NF,GAP43)*; **ELISA:** T10 SC *(BDNF)*	Perfusion (*n* = 5) **IF:** T10 SC *(CD68, Cle-caspase3/NeuN, ATF3, GFAP, GAP43, NF)*; **WB:** T10 SC *(Pro-caspase3/Cle-caspase3,PSD95,SYN,NF,GAP43)*; **ELISA:** T10 SC *(BDNF)*	Perfusion (*n* = 5) **mRNA sequencing:** SMC/T10 SC; **WB:** T10 SC *(Pro-caspase3/Cle-caspase3,PSD95,SYN,NF,GAP43)*; **ELISA:** T10 SC *(BDNF)*
**8w**	CMEPs detection; BDA anterograde tracing (*n* = 5)	CMEPs detection; BDA anterograde tracing (*n* = 5)	CMEPs detection; BDA anterograde tracing (*n* = 5)	CMEPs detection (*n* = 5)
**10w**	Perfusion (*n* = 5) **IF:** Raphe nucleus *(c-Fos/BDA/NeuN, SYN/BDA/NeuN)*; C3 SC *(c-Fos/BDA/NeuN);* T10 SC *(BDA/Map2)*; **DAB:** T10 SC *(5-HT)*	Perfusion (*n* = 5) **IF:** T10 SC *(BDA/Map2)*; **DAB:** T10 SC *(5-HT)*	Perfusion (*n* = 5) **IF:** T10 SC *(BDA/Map2)*; **DAB:** T10 SC *(5-HT)*	No perfusion

### 2.2 Spinal cord transection

**Figure 1:**
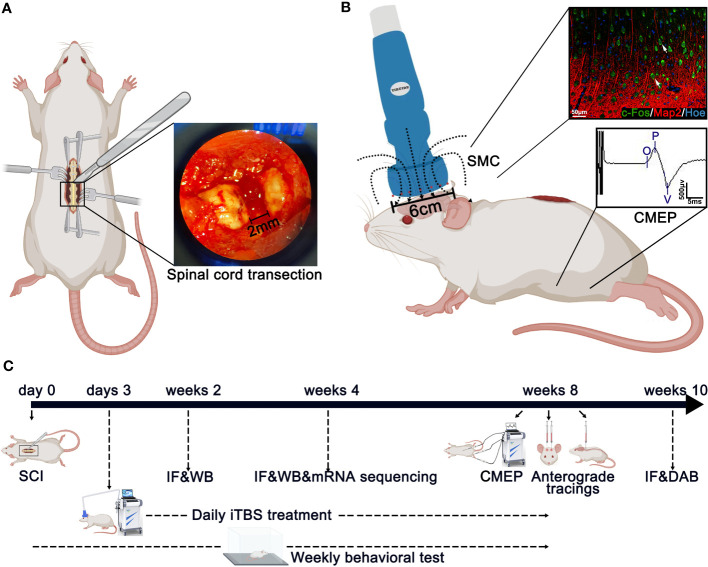
Main experimental strategy. **(A)** The method used to create spinal cord injury surgery in rats. A 2-mm cord segment was completely removed at the T10 level. **(B)** The magnetic field distribution produced by the special circular coil for rats. The expression of c-Fos was detected on the layer 5 pyramidal neurons in the SMC after iTBS in normal rats. Meanwhile, the CMEPs were recorded from the anterior tibialis after iTBS treatment in SCI rats. **(C)** Experimental paradigms illustrating the timelines of the major experimental manipulations.

The spinal cord transection model was described in our previous research (23). Rats were first anesthetized by an intraperitoneal injection (0.35 ml/100 g) of 1% pentobarbital sodium (China Pharmaceutical Shanghai Chemical Reagent Company, Shanghai, China). Then, laminectomy was performed to expose the T9 and T10 spinal cord segments, and the dura was slit vertically with a pair of micro-forceps and micro-scissors under a stereomicroscope. A 2-mm cord segment including the associated spinal roots, was completely removed at the T10 level (**Figure 1**). A gel sponge was used to stop bleeding of the spinal cord at the site of injury and soft tissues were sutured layer by layer with 4-0 surgical sutures. Following surgery, rats were placed on a heating pad until they wakened from anesthesia. After surgery, each rat was injected subcutaneously with 0.05 mg/kg of buprenorphine (Tianjin Pharmaceutical Research Institute Pharmaceutical Co., Ltd., Tianjin, China) every 12 hours for 3 consecutive days to relieve pain and also injected intramuscularly with penicillin G(50,000 U/kg/d, Jiangxi Keda Animal Pharmaceutical Co., Ltd., Jiangxi, China) to prevent infection for 7 days (26, 27). For 7 days after SCI, the rats were reared in a single cage and their bladders were massaged 3 times a day to assist urination. On day 7 after SCI, five rats were housed per cage, and urination was assisted twice daily until the rats were sacrificed.

### 2.3 Transcranial magnetic stimulation

Rats from the iTBS group were treated 72 hours after SCI with TMS (YRD CCY1; Wuhan Yiruide Medical Equipment New Technology Co., Ltd., Wuhan, China) equipped with a special circular coil for rats (r = 32 mm, Y064; Wuhan Yiruide Medical Equipment New Technology Co., Ltd., Wuhan, China) and a pulsed magnetic field with 3-T peak intensity (**Figure 1**). During treatment, the center of the magnetic field produced by the coil was placed close to the center of the skull of the rat being tested. Each rat was repeatedly stimulated 20 times a day (600 pulses in total for 200 s) for five days a week until they were sacrificed. The iTBS protocol used in this study consisted of three-pulse bursts at 50 Hz repeated at 5 Hz. A 2-s stimulation of TBS was repeated every 10 s for 20 repetitions, for a total of 600 pulses. A movement threshold (MT) of 90% was selected as the stimulus output intensity (28). Rats in the sham iTBS group were given sham stimulation with the same parameters 72 hours after SCI, although the coil was placed perpendicular to the skull.

The mean MT of rats (*n* = 55) was measured for further iTBS treatment before SCI. The recording electrode was inserted into the muscle belly of the tibialis anterior in a hindlimb; the grounding electrode was fixed on the rat’s back, and the center of the stimulation coil was placed above the skin on the center of the skull. In a non-anesthetic state, the intensity started from low-intensity stimulation that evoked ankle dorsiflexion, and CMEPs was recorded from the tibialis anterior. MT was defined as the minimum TMS intensity at which the amplitude of CMEPs was not less than 100 μV for 5 out of 10 stimulation sessions during slight muscle contraction. Pre-testing results showed that the MT for iTBS treatment was 25% maximal stimulator output.

### 2.4 Detection of cortical motor evoked potentials detection

In a non-anesthetic state, five rats in each group were tested for CMEPs at 8 weeks after SCI. CMEPs were detected with a CCY1 magnetic stimulation therapy instrument (YRD CCY1; Wuhan Yiruide Medical Equipment New Technology Co., Ltd., Wuhan, China). The sites used for electrode insertion were the same as those used for MT measurement. The center of the stimulation coil was placed above the center of the brain skull, and the output intensity was 100% maximum stimulation intensity. The amplitudes of CMEPs were recorded in the tibialis anterior of the hindlimb during stimulation of the cerebral motor cortex. Each rat was stimulated 20 times (30-s intervals) and amplitudes were recorded.

### 2.5 Behavioral testing

Double-blinded 5-min Basso–Beattie–Bresnahan (BBB) test scores were used weekly to evaluate hindlimb joint movement, walking ability, coordination, and the stability of rats after SCI (29). A modified inclined-grid climbing test was used to observe and film coordinating movements between the forelimbs and hindlimbs to evaluate the overall locomotor ability of each animal (23). Each rat was placed on a grid with a 45° slope and allowed to climb the grid three times. We recorded the total number of “pedal steps” on the grid by both hind legs.

### 2.6 Biotinylated dextran amine anterograde tracing

#### 2.6.1 BDA tracing in the SMC

Five rats were selected from each group for anterograde BDA labeling 8 weeks after SCI. The head was fixed onto the rat brain stereolocator under anesthesia; based on stereotaxic mapping of the brain, we created two round bone windows (r = 2.5 mm) at a point 1 mm anterior to the fontanelle and 1 mm from the sagittal suture bilaterally to expose the SMC in the bilateral cerebral hemispheres. Then, 10% (Molecular weight 10000) BDA solution (Invitrogen D1956; Thermo Fisher Scientific, Waltham, MA, USA) was injected slowly under a surgical microscope.

Six injection sites were selected bilaterally; these were located 1 mm, 2 mm, or 3 mm behind the anterior fontanelle and 1 mm or 2 mm from the sagittal suture. At each site, 0.25 μl was injected at a depth of 2 mm and 1 mm from the SMC surface; thus, a total of 0.5 μl was injected at each site. The needle was retained for 2 min before being slowly withdrawn.

#### 2.6.2 BDA tracing in long descending cervical propriospinal neurons

C3 and C4 are the segments where neurons in the long descending propriospinal tract (LDPT) are located. After anesthesia, a 2-cm incision was generated to fully expose and remove the C3 and C4 spinous processes and vertebral bodies. Five sites on each side were injected with 0.5 μl of 10% BDA tracer, with a total of 5 μl bilaterally. Operations were performed at a depth of 1 mm from the surface of the spinal cord, and the needle was retained for 2 min after injection. After surgery, penicillin was injected intramuscularly (50,000 U/kg/d) once a day for 5 days, and rats were sacrificed after 2 weeks of single-cage housing.

### 2.7 Perfusion and tissue preparation

For perfusion, each animal was anesthetized and a needle was inserted through the left apex of the heart into the aorta. The right atrium was immediately opened after hemostatic forceps fixation for perfusion using pre-cooled phosphate-buffered saline (PBS, 4°C, ZLI-9062; Wuxi, China) until blood was cleared from the liver.

Two weeks after SCI, five rats in each group were perfused with PBS, as described previously. Whole brains were removed by craniotomy. One side of each brain hemisphere was fixed with 4% paraformaldehyde (PFA, C10814814; Macklin, Shanghai, China) at 4°C for 3 days, followed by dehydration with 30% sucrose solution for 2 weeks. The SMC on the other side (2 mm from the coronal suture and 3 mm from the sagittal suture) was removed carefully and washed in PBS. Next, tissues were placed directly into microcentrifuge tube and immediately stored at -80°C.

Four weeks after SCI, five rats in each group were perfused with PBS. The SMC was removed carefully and washed in diethylpyrocarbonate-treated ddH2O (DEPC, R0022; Beyotime, Shanghai, China) and immediately snap-frozen in liquid nitrogen. Frozen tissues were immediately stored at -80°C. RNA extraction was then performed. In addition, 2-cm of spinal cord (with T10 as the center) was removed and fixed in 4% PFA at 4°C for 3 days. Spinal cord tissue sections in the rostral injury region were removed, placed on ice, and divided into two portions. One portion was placed directly into a microcentrifuge tube; the other portion was washed in DEPC and immediately snap-frozen in liquid nitrogen. Frozen tissues were immediately stored at -80°C.

Ten weeks after SCI, five rats in the Control group, sham iTBS group, and iTBS group, were perfused with 4% PFA until they shook their limbs and tails. The total amount perfused into each rat was approximately 100 ml. Then, 2-cm spinal cord tissue sections were removed (with T10 as the center) and fixed with 4% PFA at 4°C for 2 days. This was followed by dehydration with 30% sucrose solution for 2 weeks. The specific processes used for tissue preparation are given in **Table S1**.

### 2.8 Western blot analysis

RIPA lysis buffer (P0013; Beyotime, Shanghai, China) was mixed with protease inhibitor (05892791001; Roche, Basel, Switzerland) and added microcentrifuge tubes containing SMC tissue or spinal cord tissue. The tissue was then broken up by ultrasound, placed on ice for 20 min, and centrifuged at 12000 rpm for 20 min. The supernatant (containing protein) was then stored at -80°C.

Protein samples were diluted with 4-μl of 5X loading buffer (30405; CWBIO, Beijing, China) and ddH_2_O and made up to 20 μl with protein loading solution. The samples were then boiled in a water bath for 10 min before centrifugation. The electrophoresis apparatus (1645050; Bio-Rad, Hercules, CA, USA) was used to adjust the voltage to 80 V for 30 min until the loading buffer indicator ran out of the concentrated gel. Then, the voltage was adjusted to 100 V for 70 min until the indicator ran to the bottom of the gel. Gels were then transferred with a “sandwich” clip at a current of 250 mA for 4 hours. Next, membranes were incubated in blocking solution for 20 min and then incubated overnight at 4°C with specific primary antibodies and nutation. The following morning, membranes were placed on a shaker at room temperature for rewarming and washed with 1x Tris- buffered saline containing Tween 20 (TBST, 17322; CWBIO, Beijing, China). Next, the membranes were incubated with horseradish peroxidase (produced by the same species as the antibody) for two hours at room temperature. An electrochemiluminescence reagent (BL523A; Biosharp, Anhui, China) was then used for the chemiluminescence imaging system (ChemiDoc Touch 1708370; Bio-Rad, Hercules, CA, USA). Bands (from 5 rats per group) were analyzed by ImageJ. **Table S2** provides a summary of the antibodies used for western blotting.

### 2.9 Immunofluorescence analysis

After fixation in 4% PFA, samples of cerebral hemispheres and spinal cord were dehydrated in 30% sucrose solution for 2 weeks. The SMC and spinal cord from the lesion area were then embedded in Tissue-Tek^®^ O.C.T. (Sakura Finetek, Alphen aan den Rijn, Netherlands) and 25 μm coronal sections were cut with a cryotome. Sections were circled with a histochemical pen and sealed with 10% goat serum diluted in PBS with 0.3% Triton X-100 (EZ7890C254; BioFroxx, Beijing, China) in a thermotank at 37°C for 30 min. Samples were then incubated overnight at 4 °C with specific primary antibodies.

The next day, samples were washed three times with 0.01 mol/L of PBS (5 minutes per wash) and then incubated with secondary antibodies for 2 hours at 37°C. Cell nuclei were counterstained with Hoechst33342 (Hoe) for 15 min. Slides were imaged using a confocal microscope (Dragonfly CR-DFLY-202 2540; Andor Technology, Belfast, UK). Immunofluorescence quantitative analysis was performed on tissue sections from 5 rats per group. Three coronal sections were selected from the cerebral hemisphere of each rat and from the regions rostral and caudal to/in the injury site of the spinal cord. These sections were analyzed by positive cell counting or mean gray values in ImageJ. **Table S2** shows a list of the antibodies used for immunofluorescence analysis.

### 2.10 3,3’-diaminobenzidine staining

Tissue sections were incubated with 3% H2O2 in deionized water at room temperature to eliminate endogenous peroxidase activity. Sections were then incubated overnight at 4 °C with a primary antibody raised against 5-hydroxytryptamine (5-HT, S5545; Sigma, SL, USA) and then rewarmed for 30 min. Sections were then incubated for 30 min at 37°C with secondary antibodies and washed three times with PBS (5 min per wash). Thereafter, a streptavidin-biotin complex (SABC; SA1020; Boster, Wuhan, China) was added to sections for 30 min at 37°C. Next, the tissues were washed three times with 0.01 mol/L of PBS (5 min per wash). SABC (SA1020; Boster, Wuhan, China) was then added and incubated at 37°C for 30 min. Then, after three washes with 0.01 mol/L of PBS, we added 1 ml of liquid A and 50 µl of liquid B (SA1020; Boster, Wuhan, China) for color development. When brown fibers were visualized under the microscope, the sections were washed with ddH_2_O to stop the reaction. Then, the sections were stained with hematoxylin (C0107; Beyotime, Shanghai, China) at room temperature for 5 minutes. Sections were then washed with ddH_2_O for 1 min, decolorized with 1% hydrochloric acid alcohol for 20 seconds and then rinsed in running water for 30 min. Next, the sections were decolorized with 90%, 95% and 100% ethyl alcohol for 20 seconds, 2 min and 5 min, respectively. Then, the sections were treated twice (15 min per treatment) with xylene (Guangzhou Chemical reagent Field, Guangzhou, China) and finally sealed with neutral balsam (Shanghai Specimen and Model Field, Shanghai, China).

### 2.11 mRNA sequencing

Four weeks after SCI, three rats were selected from the normal group, the sham iTBS group, and the iTBS group, for mRNA sequencing in the SMC and spinal cord. RNA was extracted according to conventional methods. RNA integrity was determined by agarose gel electrophoresis (28S:18S ≥ 1.5), RNA purity was detected using a NanoDrop spectrophotometer (optical density [OD]260/280: 1.8–2.2), and RNA concentration was accurately quantified using a Qubit fluorometer (≥ 500 ng/μl). Samples were sent to Genergy Bio-Technology Co., Ltd. (Shanghai, China) for RNA-Seq analysis. Data generated by a high-throughput sequencer were then converted into sequenced reads by CASAVA base calling. The original sequence counts of known genes for all samples were analyzed by StringTie, and the expression levels of known genes were calculated by fragments per kilobase of transcript per million fragments mapped. DESeq2 software was used to identify differentially expressed genes (DEGs) between groups, with |log2(fold change [FC]) | values ≥ 1 and *P*-values ≤ 0.05.

### 2.12 Statistical analysis

Data are expressed as the mean ± Standard error (SEM). Data were analyzed by one-way analysis of variance (ANOVA) in GraphPad Prism software (v 8.0; GraphPad Software, San Diego, CA, USA) for normal distributions of variable values, and Dunnett’s *post hoc* test was used when the variances were not uniform. For the non-normal distribution of variable values, Kruskal-Wallis test was used to analyze the differences between groups. For all experiments, values of *P* < 0.05 were considered to be statistically significant.

## 3 Results

### 3.1 Effects of transcranial iTBS on inflammation and neuronal apoptosis in the SMC

**Figure 2:**
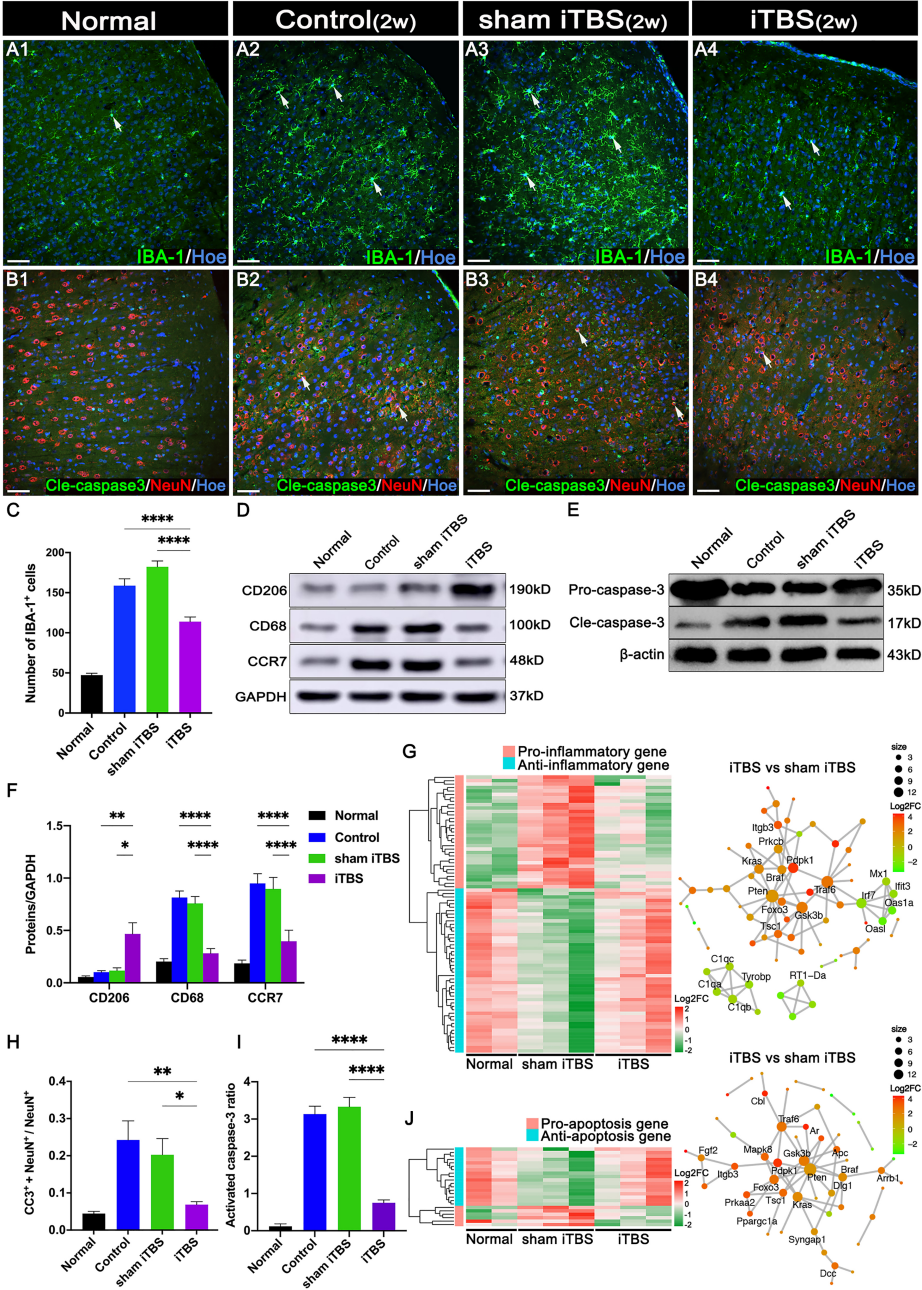
Effects of transcranial iTBS on inflammation and neuronal apoptosis in the SMC. **(A)** IBA-1^+^ microglia/macrophages in each group two weeks after SCI. **(B)** The co-expression of Cle-caspase3 and NeuN in each group. **(C)** Bar chart showing the number of IBA-1^+^ cells in each group (****P < 0.0001). **(D)** Western blot showing CD206, CD68, CCR7, and GAPDH in the SMC. **(E)** Western blot showing Pro-caspase3, Cle-caspase3, and β-actin in the SMC. **(F)** Bar chart showing the quantification of the protein expression based on western blots in each group (****P < 0.0001, **P < 0.01, *P < 0.05). **(G)** Heatmap constructed from 81 up-regulated (red) and down-regulated (green) expressed genes with significant differences related to inflammation in the SMC by clustering analysis. The network diagram among genes between the neuronal activation and inflammation sets by enrichment analysis was constructed in the iTBS group *vs.* the sham iTBS group. Based on enrichment analysis of the functional sets between the neuronal activation and inflammation sets in the iTBS group *vs.* the sham iTBS group, we constructed a network diagram among genes inside these two functional sets. **(H)** Bar chart showing the relative expression of cle-caspase3 (CC3^+^) in NeuN^+^ cells in each group (**P < 0.01, *P < 0.05). **(I)** Bar chart showing quantification of the protein expression of activated caspase3 (Cle-caspase3/Pro-caspase3) based on western blots in each group (****P < 0.0001). **(J)** Heatmap constructed from 23 up-regulated (red) and down-regulated (green) genes with significant differences related to apoptosis in the SMC by clustering analysis. A network diagram among genes between the neuronal activation and apoptosis sets by enrichment analysis was constructed in the iTBS group *vs.* the sham iTBS group. Cell nuclei were counterstained with Hoechst33342 (Hoe). The network was extracted using the STRING database for rats (confidence coefficient ≥ 700), and each node is shown in color according to the Log2FC value. Data are presented as mean ± SEM (n = 5). Scale bars = 50 μm in (A1–B4).

Transcranial iTBS treatment was commenced 72 hours after SCI. Layer 5 pyramidal neurons in the SMC were successfully activated to express c-Fos and hindlimb CMEPs were successfully recorded(**Figure 1**). Transcranial iTBS treatment significantly reduced the expression of the microglia/macrophage marker ionized Ca^2+^-binding adapter protein 1 (IBA-1) in the SMC when determined 2 weeks after SCI (**Figures 2A, C**). To further evaluate the effects of transcranial iTBS on inflammation, we analyzed tissue obtained from the SMC by western blotting to detect alterations in microglia/macrophage-associated proteins. Analysis showed that the levels of macrosialin (CD68) and C-C chemokine receptor 7 (CCR7, an M1 macrophage marker) were significantly reduced after 2 weeks of transcranial iTBS (**Figures 2D, F**). Furthermore, transcranial iTBS increased the expression levels of the mannose receptor (CD206, an M2 macrophage marker) after SCI (**Figures 2D, F**). Moreover, after 2 weeks of transcranial iTBS treatment, we found that the number of apoptotic neurons co-labeled by Cleaved caspase 3 (Cle-caspase3) and NeuN was significantly lower than that in the Control and sham iTBS groups. There were no significant differences between the Control and sham iTBS groups; similar results were produced by western blot analysis (**Figures 2B, H, E, I**). Differential gene expression related to inflammation and apoptosis further supported these results. The expression levels of 33 pro-inflammatory genes and 6 pro-apoptosis genes in the SMC after 4 weeks of transcranial iTBS were significantly lower than those in the sham iTBS group. Moreover, compared with the sham iTBS group, the expression levels of 48 anti-inflammatory genes and 17 differentially expressed genes that promote cell survival and inhibit apoptosis were more similar to those in the Normal group (**Figures 2G, J**; **Table S3**). The expression level of the immediate early gene c-Fos is known to reflect the activation of neurons. We found that transcranial iTBS up-regulated the expression of c-Fos in neurons in the SMC when compared with the Control group in normal rats (**Figures S1A, D**).

### 3.2 Effects of transcranial iTBS on inflammation and neuronal apoptosis at the site of SCI

**Figure 3:**
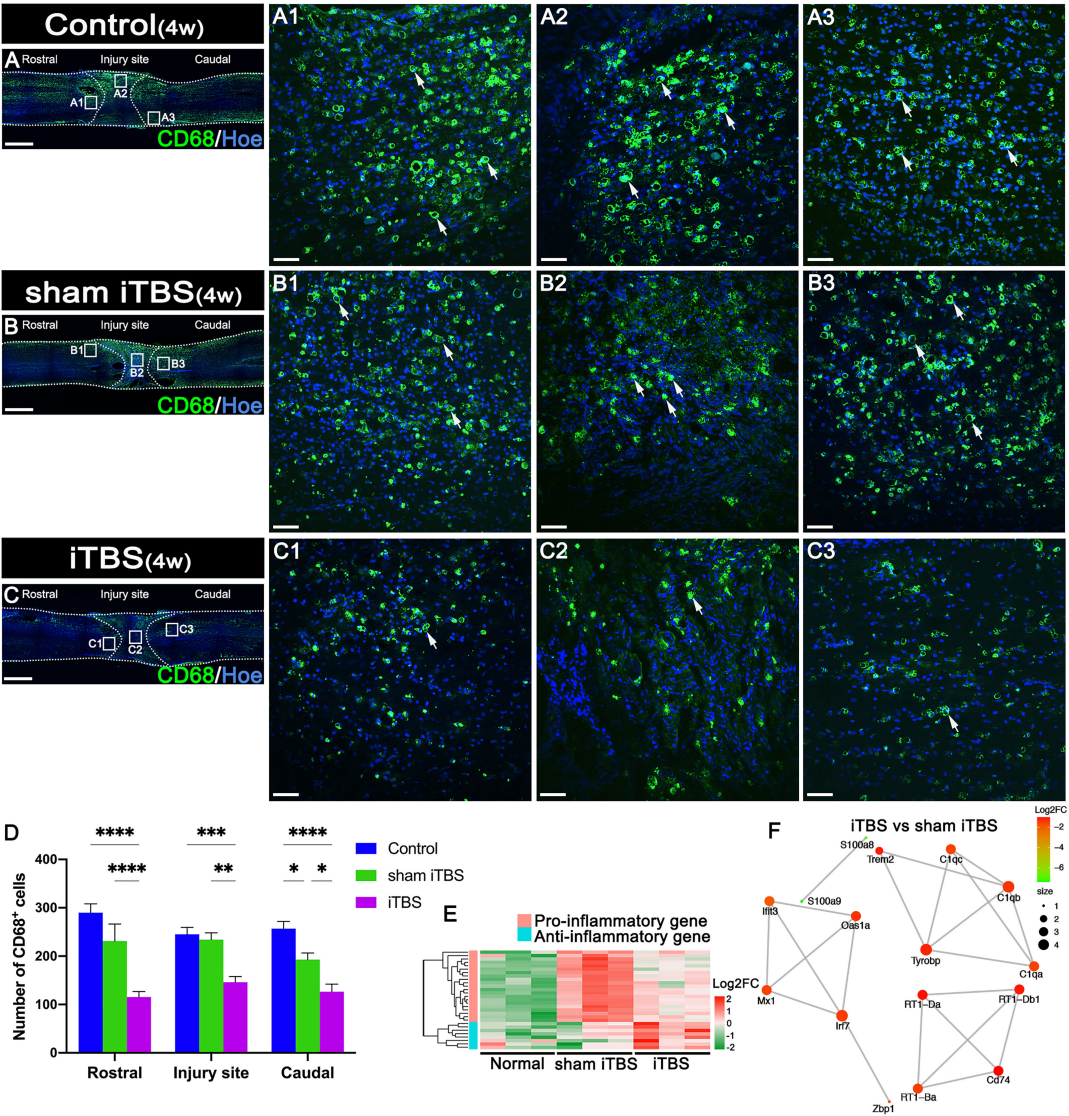
Effects of transcranial iTBS on inflammation in the injury site of spinal cord. **(A–C)** Low magnification of coronal spinal cord sections showing the expression of CD68. (A1–C3) The pictures are the magnification of the area indicated in the box area of **(A–C)**. Arrows indicate CD68^+^ cells in green. **(D)** Bar chart showing the number of CD68^+^ cells in the regions rostral and caudal to/in the injury site (****P < 0.0001, ***P < 0.001, **P < 0.01, *P < 0.05). **(E)** Heatmap constructed from 29 significantly differentially expressed genes related to inflammation in the rostral injury region. Following clustering analysis, up-regulated and down-regulated genes are colored in red and green, respectively. **(F)** Based on enrichment analysis of the functional sets between the neuronal activation and inflammation sets in the iTBS group *vs.* the sham iTBS group, we constructed a network diagram among genes inside these two functional sets. The network was extracted using the STRING database for rats (confidence coefficient ≥ 700) and each node is shown in color according to the Log2FC value. Cell nuclei were counterstained with Hoechst33342 (Hoe). Data are presented as mean ± SEM (n = 5). Scale bars =1000 μm in **(A–C)**; 50μm in (A1–C3).

**Figure 4:**
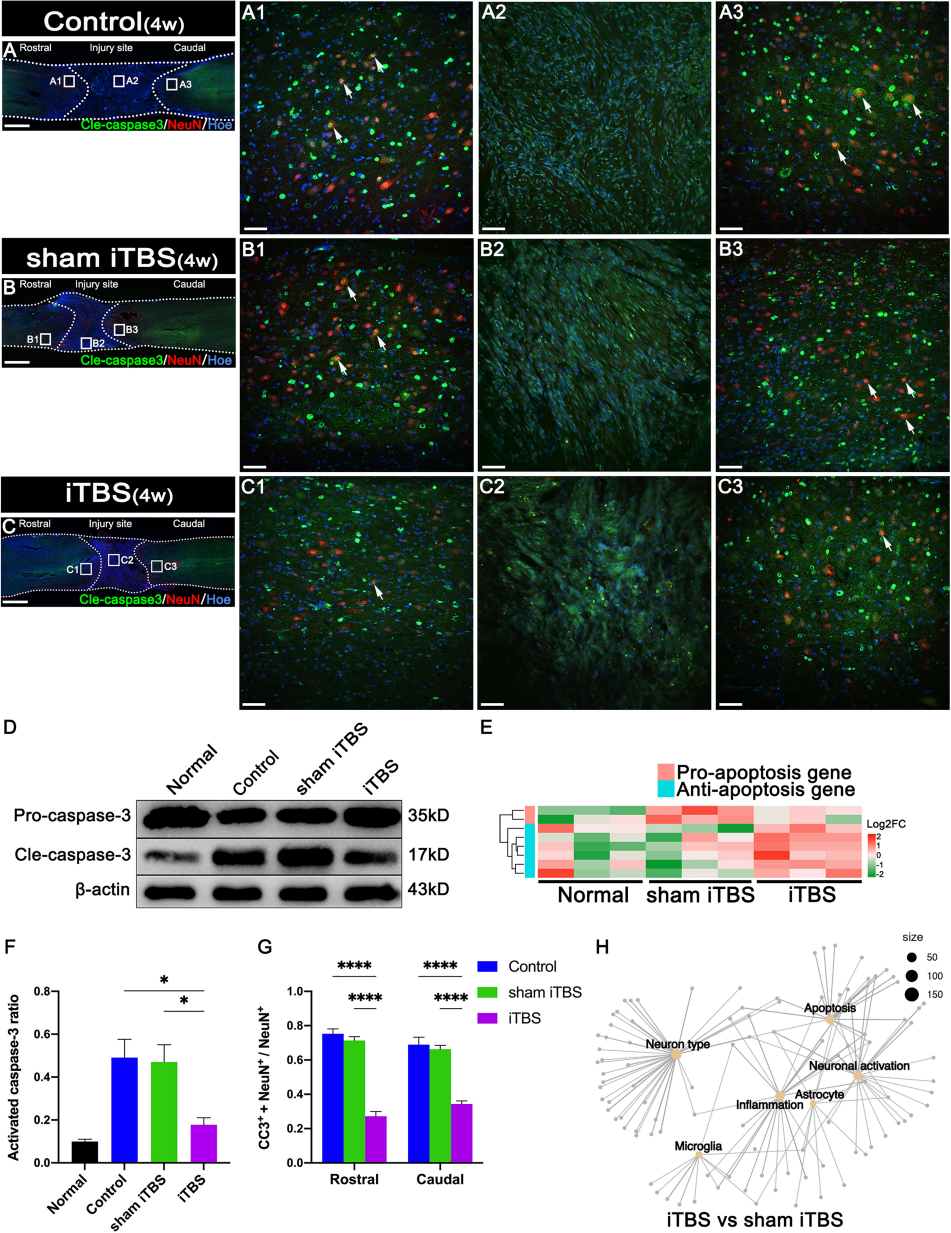
Effects of transcranial iTBS on neuronal apoptosis in the injury site of spinal cord. **(A–C)** Low magnification images of coronal spinal cord sections showing the expression of Cle-caspase3 and NeuN. (A1–C3) The pictures are the magnification of the area indicated in the box area of **(A–C)**. Arrows indicate cle-caspase3^+^ neurons. **(D)** Western blot for Pro-caspase3, Cle-caspase3, and β-actin in the rostral injury region. **(E)** Heatmap constructed from 8 significantly differentially expressed genes related to apoptosis in the rostral injury region. Following clustering analysis, up-regulated and down-regulated genes are colored in red and green, respectively. **(F)** Bar chart showing quantification of the protein expression of activated caspase3 (Cle-caspase3/Pro-caspase3) based on western blotting (*P < 0.05). **(G)** Bar chart showing the relative expression of cle-caspase3 (CC3^+^) in NeuN^+^ neurons in the regions rostral and caudal to the injury site (****P < 0.0001). **(H)** Network diagram showing the association of enriched genes in the iTBS group when compared with the sham iTBS group in six different functional sets. The node size of the functional set represents the total number of candidate genes by gene ontology. Cell nuclei were counterstained with Hoechst33342 (Hoe). Data are presented as mean ± SEM (n = 5). Scale bars =1000 μm in **(A–C)**; 50 μm in (A1–C3).

After 4 weeks of transcranial iTBS treatment, the expression levels of CD68 in the regions rostral and caudal to/in the injury site of the spinal cord in the iTBS group were significantly lower than those in the Control and sham iTBS groups (**Figures 3A–D**). We also observed a significant reduction in the number of neurons co-expressing Cle-caspase3^+^ and NeuN^+^ neurons in the rostral and caudal injury area (arrows in **Figures 4A–C, G**); this was also confirmed by western blotting involving tissues from the rostral injury area (**Figures 4D, F**). Next, we investigated changes of gene expression in the rostral injury region after 4 weeks of transcranial iTBS treatment. We found that 21 differentially expressed inflammatory genes and 6 differentially expressed apoptosis genes were down-regulated (**Figures 3E**, **4E**; **Table S4**); these trends were similar to the expression levels seen in the Normal group. Transcranial iTBS also upregulated the expression of c-Fos in neurons located in the cervical and thoracic spinal cord when compared with the Control group in normal rats (**Figures S1B, C, E, F**). Based on the genes related to neuronal activation and inflammation, we generated a network to represent the relationships between genes in these two functional sets (**Figure 3F**). In the network, the expression levels of *S100a9* and *S100a8* in the iTBS group were significantly lower than those in the sham iTBS group (30). The gene ontology interaction network also showed that transcranial iTBS activated spinal cord neurons and played an important regulatory role in the activation of astrocytes and microglia/macrophage (**Figure 4H**).

### 3.3 The neuroprotective effects of transcranial iTBS at the site of SCI

**Figure 5:**
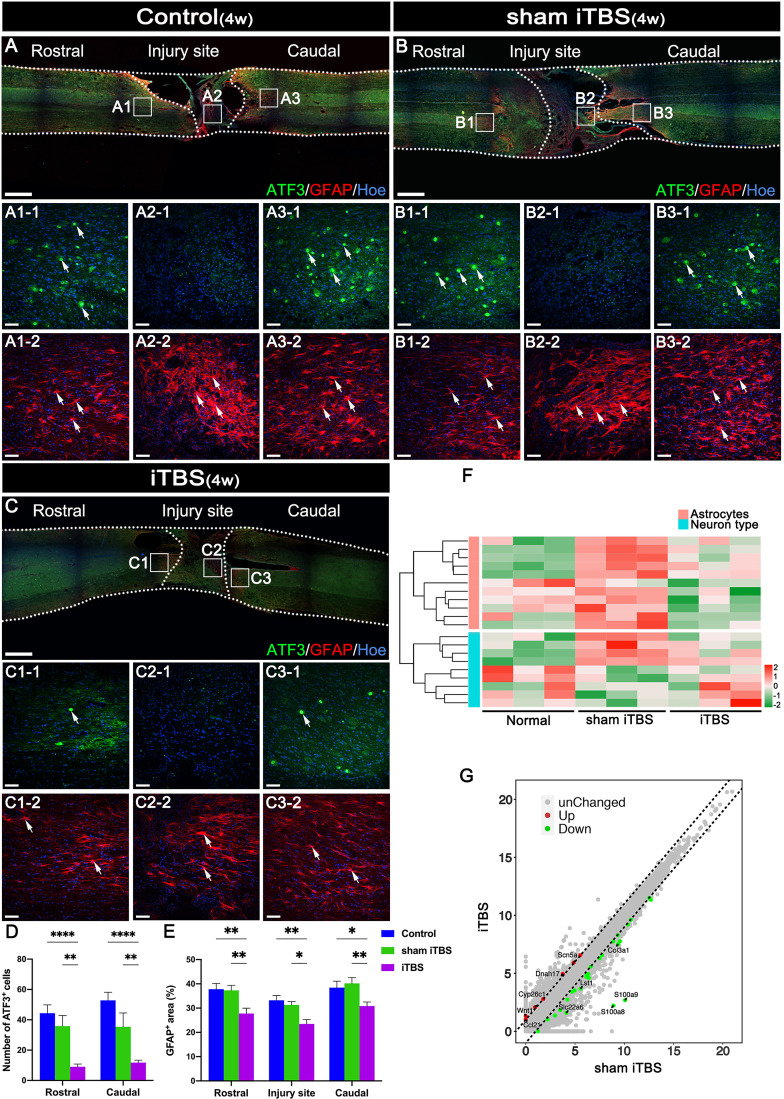
The neuroprotective effect of transcranial iTBS on the spinal cord injury site. **(A–C)** Low magnification images of coronal spinal cord sections showing the expression of ATF3 and GFAP. (A1-1-C3-1) Magnified images of the area indicated in the boxed area in **(A–C)**. Arrows indicate ATF3^+^ cells in green. (A1-2-C3-2) Magnified images of the boxed area show in **(A–C)**. Arrows indicate GFAP^+^ cells in red. **(D)** Bar chart showing the number of ATF3^+^ cells in the regions rostral and caudal to the injury site (****P < 0.0001, **P < 0.01). **(E)** Bar chart showing the relative density of GFAP^+^ cells in the regions rostral and caudal to/in the injury site (**P < 0.01, *P < 0.05). **(F)** Heatmap constructed from 20 significantly differentially expressed genes related to types of neurons and astrocytes in the region rostral to the injury site in the spinal cord. Following clustering analysis, up-regulated and down-regulated genes are colored in red and green, respectively. **(G)** Each point in the scatter plot represents a gene. The abscissa represents the gene expression levels in the sham iTBS group and the ordinate represents the gene expression levels in the iTBS group. Therefore, the genes significantly up-regulated in the iTBS group compared with the sham iTBS group are shown in red, the genes significantly down-regulated in the iTBS group compared with the sham iTBS group are shown in green, and the gray dots represent genes with no significant difference. Cell nuclei were counterstained with Hoechst33342 (Hoe). Data are presented as mean ± SEM (*n* = 5). Scale bars =1000 μm in **(A–C)**; 50 μm in (A1–C3).

Following SCI by transection, astrocytes labeled by neuroglial fibrillary acidic protein (GFAP) surrounding the lesion developed hypertrophy and protrusions (Figures 5A-2, B-2, C-2). These reactive astrocytes migrated towards the central injury site in the spinal cord (**Figures 5A2-2, B2-2, C2-2**) and are known to eventually form glial scars that secrete axon growth inhibitors and prevent axon regeneration (31). Notably, after 4 weeks of transcranial iTBS treatment, the GFAP^+^ area at the site of injury in the spinal cord was significantly reduced (**Figure 5E**), thus alleviating the adverse effects on axonal regeneration (**Figure 5F**) (**Table S4**). In addition, *activating transcription factor 3* (*ATF3*) is known to be one of the earliest genes to respond to axonal injury (32). After SCI, *ATF3* was not expressedat the central site of injury (**Figures 5A2-1, B2-1, C2-1**). However, the number of ATF3^+^ cells in the regions rostral and caudal to the injury site was significantly increased (**Figures 5D, A1-1, A3-1, B1-1, B3-1, C1-1, C3-1**). After 4 weeks of transcranial iTBS treatment, the gene expression in different types of neurons in the iTBS group was most similar to that in the Normal group (**Figure 5F**). Transcranial iTBS significantly up-regulated neurotrophic factors and receptor-related genes and down-regulated the genes that inhibit nerve regeneration (**Figure 5G**; **Figure S4** and **Table S4**).

### 3.4 Effects of transcranial iTBS on nerve fiber regeneration and synaptic plasticity

**Figure 6:**
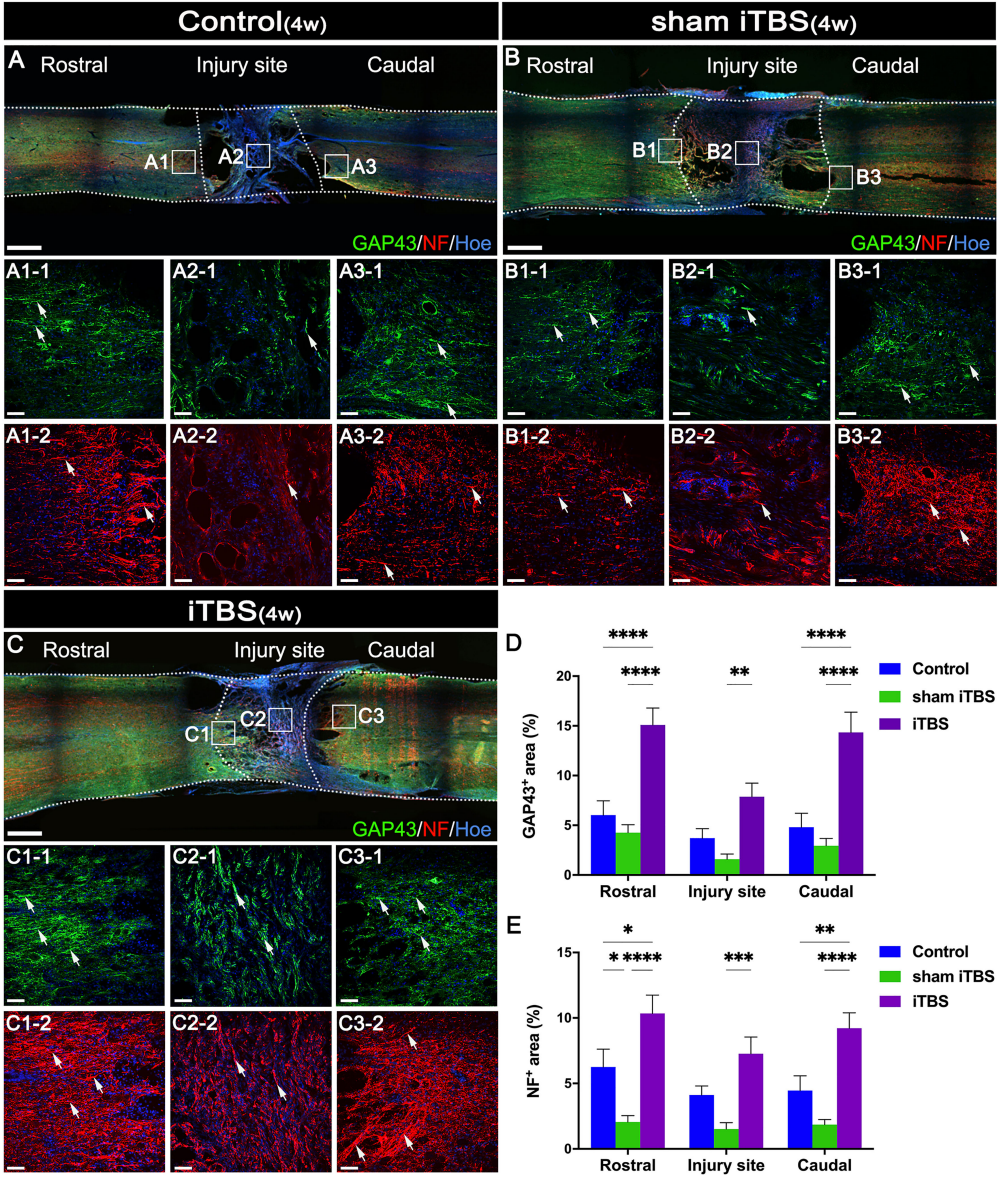
Effects of transcranial iTBS on nerve fiber regeneration and synaptic plasticity. **(A–C)** Low magnification of coronal spinal cord sections showing the expression of GAP43 and NF. (A1-1-C3-1) The pictures are the magnification of the area indicated in the box area of **(A–C)**. Arrows indicate GAP43^+^ nerve fibers in green. (A1-2-C3-2) The pictures are the magnification of the area indicated in the box area of **(A–C)**. Arrows indicate NF^+^ nerve fibers in red. **(D)** Bar chart showing the area of GAP43^+^ nerve fibers in the regions rostral and caudal to/in the injury site (****P < 0.0001, **P < 0.01). **(E)** Bar chart showing the area of NF+ nerve fibers in the regions rostral and caudal to/in the injury site (****P < 0.0001, ***P  < 0.001,**P  < 0.01, *P  < 0.05). Cell nuclei were counterstained with Hoechst33342 (Hoe). Data are presented as mean ± SEM (n = 5). Scale bars =1000μm in **(A–C)**; 50μm in (A1–C3).

To evaluate nerve fiber regeneration in a quantitative manner, we measured the fluorescence intensity of neurofilament-positive (NF^+^) nerve fibers in the regions rostral and caudal to/in the injury site. The iTBS group showed an increased area of NF^+^ nerve fibers in comparison to the Control and sham iTBS groups (**Figures 6A−C, E**).

**Figure 7:**
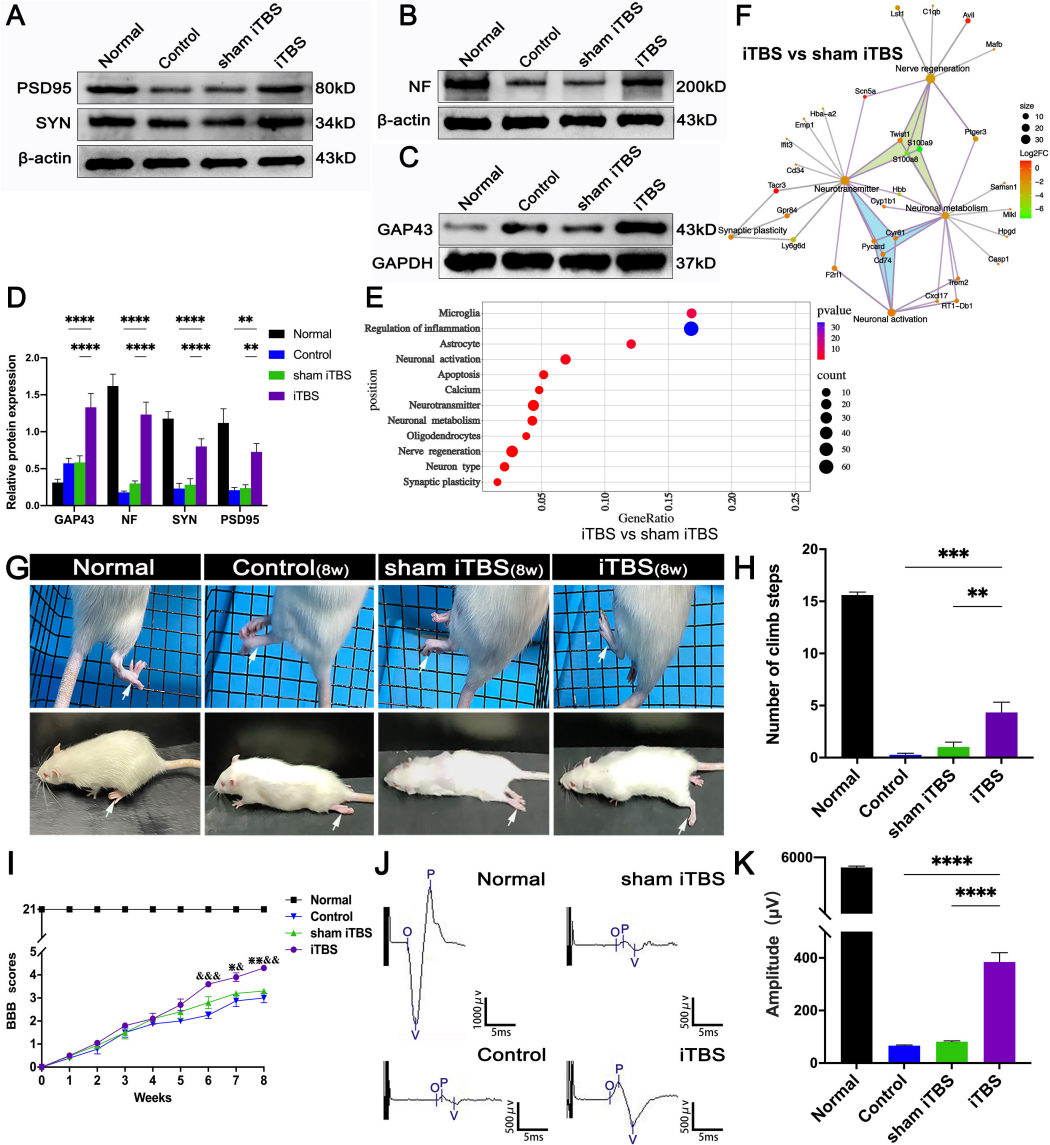
Effects of transcranial iTBS on nerve fiber regeneration and synaptic plasticity in the injury site of spinal cord and motor function after SCI. **(A)** Western blot for PSD95, SYN, and β-actin in the area rostral to injury. **(B)** Western blot for NF and β-actin in the rostral injury region. **(C)** Western blot for GAP43 and GAPDH in the area rostral to injury. **(D)** Bar chart showing quantification of protein expression based on western blotting (****P < 0.0001, **P < 0.01). **(E)** Gene ontology enrichment bubble diagram representing the expression levels of different gene sets in the iTBS group when compared with the sham iTBS group. The abscissa represents the Rich Factor (the ratio of the total number of candidate genes to the total number of all genes belonging to this gene ontology). The larger the Rich Factor, the greater the degree of enrichment. The ordinate represents different functional sets and the color represents -Log10 (*P*-value). **(F)** The node size of the function set represents the total number of candidate genes belonging to gene ontology; individual nodes between functional sets represents individual genes and the color represents the Log2FC value. **(G)** The grid climbing test and BBB assessment were performed for each group eight weeks after SCI. **(H)** Bar charts of the number of steps climbed in each group (***P < 0.001, **P < 0.01). **(I)** Comparison of BBB score for hindlimb locomotor function in each group. (^&,⋇^ indicate statistical significance when compared with the Control and sham iTBS groups, respectively, *P* < 0.05; ^&&^ indicates *P* < 0.01; ^⋇⋇^ indicates *P* < 0.01; ^&&&^ indicates *P* < 0.001). **(J)** CMEPs were obtained by electrophysiological analysis in each group 8 weeks after SCI. **(K)** Bar charts of the CMEP amplitudes in each group (****P < 0.0001). Data are presented as mean ± SEM (*n* = 5).

Changes in synaptic morphology are caused by neuronal differentiation during development of the nervous system and nerve fiber regeneration and neural repair after injury can lead to the increased expression of GAP43 (33). Compared with the Control and sham iTBS groups, the iTBS group showed that the area of GAP43^+^ nerve fibers was increased after 4 weeks of SCI (**Figures 6A–D**). Furthermore, our results showed that NF, GAP43, and the proteins related to synaptic formation, including synapsin (SYN) and postsynaptic density 95 (PSD95), were significantly decreased in the Control and sham iTBS groups when compared with the Normal group, whereas the iTBS group showed significantly higher expression levels of SYN and PSD95 than the other groups (**Figures 7A–D**).

In the gene ontology enrichment bubble diagram (**Figure 7E**), compared with the sham iTBS group, the iTBS group had a higher degree of enrichment for mRNAs associated with neuronal activation, neurotransmitters, neuronal metabolism, axonal regeneration, and synaptic plasticity. Transcranial iTBS down-regulated genes that negatively regulated neurotransmitters and neuronal metabolism and inhibited nerve regeneration through a gene interaction regulatory network (**Figure 7F** and **Table S4**). In contrast, we did not observe a relationship between neuronal activation, neuroinflammation, neuronal apoptosis, axonal regeneration, and neuroplasticity in the gene interaction network formed when comparing between the sham iTBS and iTBS groups (**Figure S5**).

### 3.5 Effects of transcranial iTBS on motor function after SCI

We performed behavioral observations in all SCI rats, including the modified grid climbing test, BBB scoring, and electrophysiological examinations, to assess the recovery of motor function after transcranial iTBS treatment. After 8 weeks of SCI, there was no placement reflex in the Control and sham iTBS groups. When the forelimbs climbed the diagonal grid, the hindlimbs were dragged behind and often fell (**Figure 7G**). In contrast, rats in the iTBS group generally showed pronounced placement reflexes in their hindlimbs, and their hind feet would occasionally tread on the grid (**Figure 7H**; **Additional Files 1**, **2**: **Videos S1**, **S2** showing the grid climbing test). Finally, BBB assessments showed that all rats suffered from complete hindlimb paralysis after T10 spinal cord transection. Compared with the Control and sham iTBS groups, the iTBS group showed significant improvement in hindlimb motor function beginning 6 weeks after treatment, the hind hips, knees, and ankles could move slightly at 8 weeks (**Figures 7G, I**; **Additional Files 3**, **4**: **Videos S3**, **S4** showing open-field locomotor test). Compared with the Control and sham iTBS groups, transcranial iTBS treatment significantly increased the response amplitude of CMEPs (**Figures 7J, K**).

### 3.6 Effects of transcranial iTBS on regeneration of corticospinal tract (CST) and 5-HT positive axons

**Figure 8:**
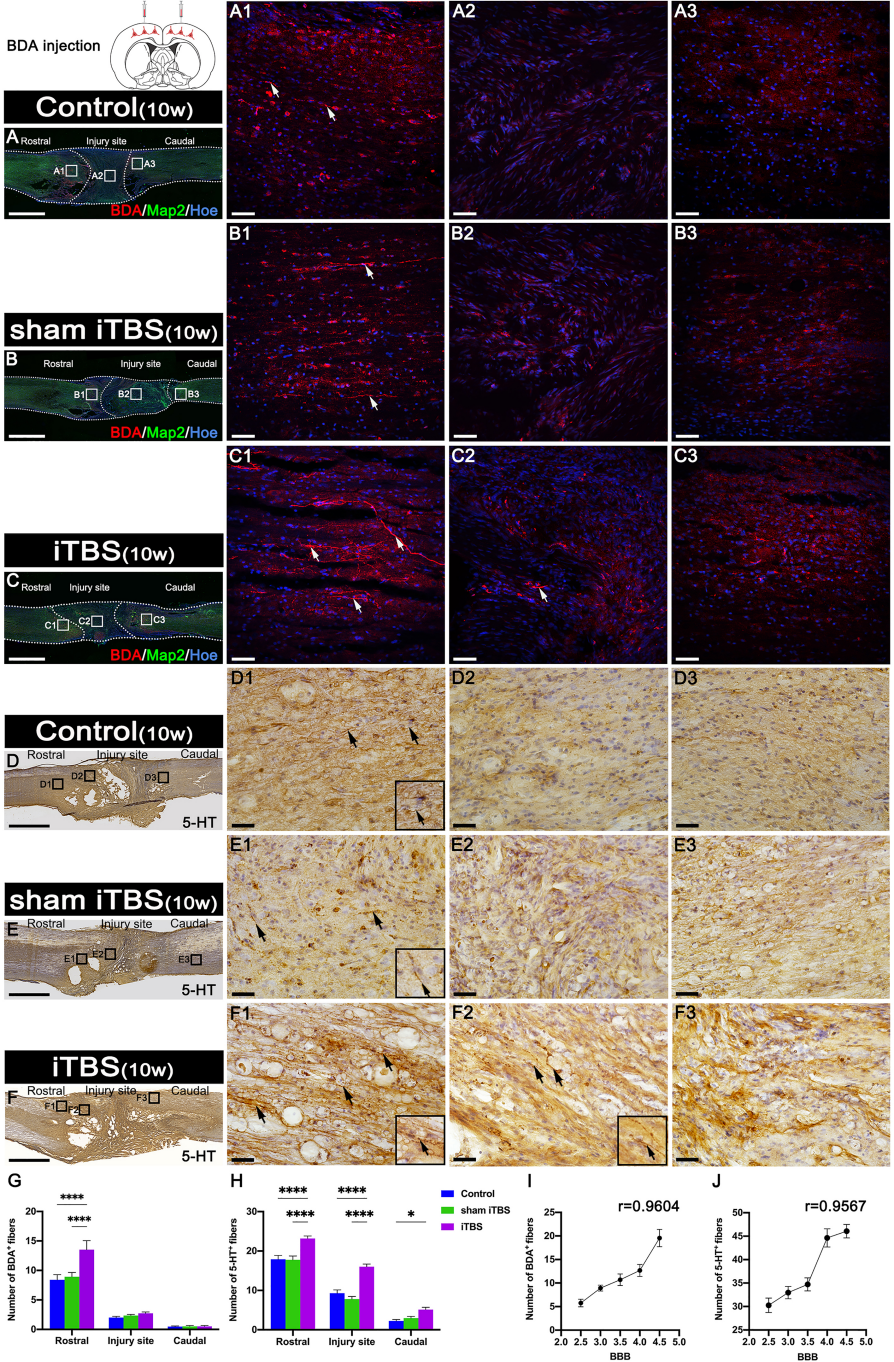
Effects of transcranial iTBS on regeneration of CST nerve fibers and 5-HT positive nerve fibers. **(A–C)** Lower magnification displaying sagittal spinal cord sections labeled by BDA. (A1-C3) The pictures are the magnification of the area indicated in the box area of **(A–C)**. Arrows indicate BDA^+^ nerve fibers in red. **(D–F)** Low magnification of sagittal spinal cord sections showing the expression of 5-HT. (D1-F3) The pictures are the magnification of the area indicated in the box area of **(D–F)**. Arrows indicate 5-HT^+^ nerve fibers. Inserts in the lower right corner of the images are the magnification of the area indicated by the arrow. **(G)** Bar chart showing the number of BDA^+^ nerve fibers in the regions rostral and caudal to/in the injury site (****P < 0.0001). Data are presented as mean ± SEM (*n* = 3). **(H)** Bar chart showing the number of 5-HT^+^ nerve fibers in the regions rostral and caudal to/in the injury site (****P < 0.0001, *P < 0.05). Data are presented as mean ± SEM (*n* = 5). **(I)** Line chart showing the linear relationship between the number of rostral BDA^+^ nerve fibers and BBB score (r = 0.9604, **P < 0.01). **(J)** Line chart showing the linear relationship between the number of rostral 5-HT^+^ nerve fibers and BBB score (r = 0.9567, *P < 0.05). Cell nuclei were counterstained with Hoechst33342 (Hoe). Scale bars =2000μm in **(A–F)**; 50μm in (A1–C3); 25μm in (D1–F3).

Fourteen days after we injected BDA into the SMC, we counted the number of BDA^+^ CST nerve fibers larger than 50 μm in length. We found that BDA^+^ nerve fibers appeared more in the rostral region to the injury site of the spinal cord in the iTBS group when compared with the Control and sham iTBS groups (**Figures 8A–C, G**). Moreover, BDA^+^ nerve fibers were mainly distributed in the dorsal deep white matter area near the midline and arranged in a relatively orderly manner and parallel to the longitudinal axis of the spinal cord in the iTBS group (**Figure 8C1**). Some disordered and enlarged ends of BDA^+^ nerve fibers were observed at the injury site (**Figure 8C2**), while no BDA^+^ nerve fibers were found in the caudal injury area (**Figure 8C3**). However, there were fewer and more disordered BDA^+^ nerve fibers in the rostral region of the injury site in the Control and sham iTBS groups (**Figures 8G, A1, B1**); no BDA^+^ nerve fibers were found in the regions central and caudal to/in the injury site (**Figures 8A2, A3, B2, B3**).

**Figure 9:**
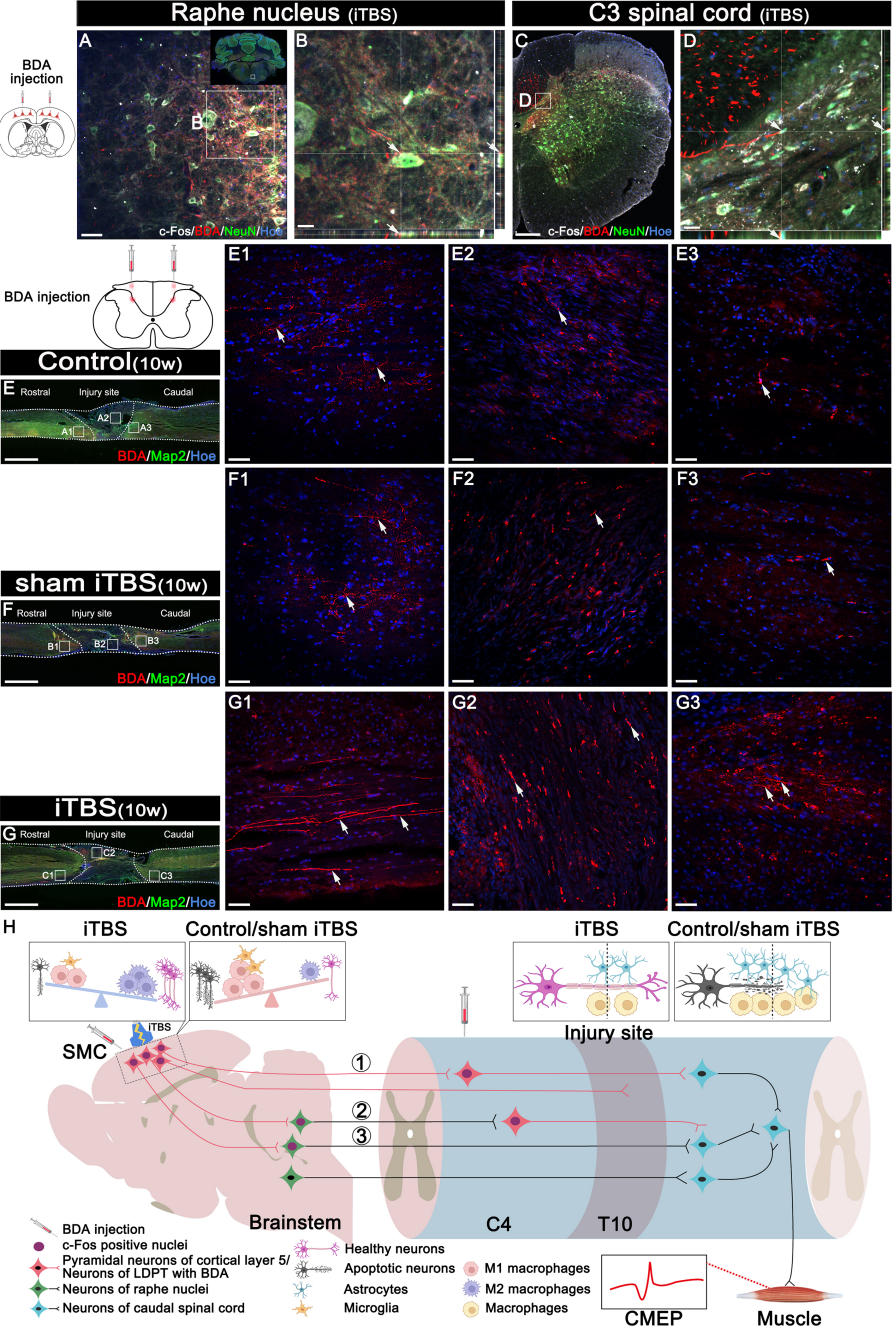
Effects of transcranial iTBS on neuron activation and regeneration of LDPT. **(A)** The co-expression of BDA and c-Fos in NeuN^+^ neurons in the raphe nucleus in the iTBS group. **(B)** The picture is the magnification of the area indicated in the box area of **(A)**. **(C)** The co-expression of BDA and c-Fos in NeuN^+^ neurons at C3 level of spinal cord in the iTBS group. **(D)** The picture is the magnification of the area indicated in the box of **(C)**. **(E-G)** Lower magnification displaying sagittal spinal cord sections labeled by BDA. (E1-G3) The pictures are the magnification of the area indicated in the box area of **(E–G)**. Arrows indicate LDPT labeled with BDA in red. **(H)** A schematic diagram of reconstructing descending motor neural pathways. The numbers represent neural pathways including: ①CST–LDPT pathway; ②CST–5-HT–LDPT pathway; ③CST–5-HT pathway. Cell nuclei were counterstained with Hoechst33342 (Hoe). Scale bars = 50 μm in **(A)** and (E1–G3); 20μm in **(B)** and **(D)**; 200μm in **(C)**; 2000 μm in **(E–G)**.

In addition, we used DAB staining to visualize the descending 5-HT^+^ nerve fibers 10 weeks after SCI (**Figures 8D–F, H**). Although 5-HT^+^ nerve fibers reached the rostral region of the injury site in the spinal cord (**Figures 8D1, E1, S3A1, B1**), fewer 5-HT^+^ nerve fibers crossed the injury site in the Control and sham iTBS groups (**Figures 8D2, E2, D3, E3, S3A2, B2, A3, B3**) than the iTBS group. Remarkably, a large number of 5-HT^+^ nerve fibers were observed in the rostral region to/in the injury site in the iTBS group (**Figures 8F1**, F2, S3C1, S3C2), but fewer 5-HT^+^ nerve fibers regenerated into the caudal region to the injury site (**Figures 8F3, H, S3C3**). The positive relationships between the number of BDA^+^ nerve fibers, the number of 5-HT^+^ nerve fibers, and BBB scores were identified (**Figures 8I, J**). Transcranial iTBS stimulated the expression of c-Fos in the raphe nucleus neurons of the brain stem (**Figures 9A, B**). More convincingly, the injection of BDA into the SMC induced synapse formation with neurons in the raphe nucleus in rats treated with transcranial iTBS (**Figure S2**). These results suggested that transcranial iTBS could activate SMC neurons and affect 5-HT neurons in the raphe nucleus to promote the regeneration of 5-HT^+^ nerve fibers and lead to the recovery of motor function.

### 3.7 Effects of transcranial iTBS on regeneration of the long descending nerve fibers of the cervical propriospinal neurons

Transcranial iTBS could stimulate the expression of c-Fos in the cervical propriospinal neurons, and some of the BDA^+^ CST fibers were shown to have made close contacts with these neurons (**Figures 9C, D**). To further explore the specific mechanism by which transcranial iTBS can improve motor function, rats in each group were injected with BDA (8 weeks after SCI) into the cervical propriospinal neurons that project long descending nerve fibers (propriospinal tract). In the iTBS group, some nerve fibers labeled with BDA were identified in the rostral injury region; furthermore, BDA^+^ nerve fibers were arranged parallel to the longitudinal axis of the spinal cord (**Figure 9G1**). Moreover, we also identified BDA^+^ nerve fibers at the center of the injury site (**Figure 9G2**). Most significantly, a small number of BDA^+^ nerve fibers were observed in the caudal injury regions (**Figure 9G3**). However, compared with the iTBS group, fewer BDA^+^ nerve fibers were observed in the rostral injury region in the Control group (**Figure 9E1**) and sham iTBS group (**Figure 9F1**). We only detected a few BDA^+^ nerve fibers in the caudal region (**Figures 9E3, F3**).

## 4 Discussion

SCI leads to the rupture of brain-derived nerve fibers, causing neuronal apoptosis and further loss of motor function. The regeneration of brain-derived nerve fibers and the reconstruction of motor pathways is the most difficult problem in SCI repair (1, 24). Previous treatments for SCI paid more attention to neuroprotection and axonal regeneration in the lesion area (23–25). However, in this study, we focused on exploring the therapeutic effects and mechanisms of transcranial iTBS on the SMC with regards to neuroprotection, axonal regeneration, and voluntary motor function reconstruction in acute, subacute, and chronic stages of complete spinal cord transection in a rat model.

### 4.1 Neuroprotective effects of transcranial iTBS on SMC

Many previous studies suggested that SCI could trigger a series of traumatic cascade reactions in the brain which manifest as inflammation and lead to the progression of neurological dysfunction (5–8, 34). Our results suggested that microglia/macrophages were the dominant immune cells in the SMC two weeks after transection SCI. Furthermore, SCI induced an increase in the number and size of microglia/macrophages which also exhibited increased expression levels of CD68 and CCR7, thus suggesting that they differentiated into the M1 phenotype and participated in the secretion of pro-inflammatory factors to trigger inflammation. However, transcranial iTBS treatment of the SMC significantly reduced the number of microglia/macrophages. We detected the expression of CD206, a marker of the M2 phenotype, thus suggesting that transcranial iTBS had a positive role in regulating the immune balance of the brain. Previous studies suggested that SCI could cause atrophy and apoptosis in SMC neurons; however, our investigations mostly focused on the chronic stage (i.e., two months after SCI) (5, 7, 8). Our results demonstrated that SMC neurons began to undergo apoptosis two weeks after SCI, and that the number of apoptotic neurons was consistent with the number of microglia/macrophages, thus suggesting that the progression of brain neuroinflammation was closely related to the apoptosis of brain neurons. It has been reported that neurons in the brain send out nociceptive signal safter SCI to induce the aggregation and activation of microglia/macrophages before neuronal apoptosis (35, 36). Our results suggested that the timely control of microglia/macrophage activation by transcranial iTBS was important in terms of preventing the programmed apoptosis of neurons.

There are few preclinical and clinical studies on the application of transcranial iTBS in SCI, and most previous SCI models involved incomplete transection (9, 17). Therapeutic effects have mainly been directed at the rehabilitation of motor function and the neuroplasticity of the spinal cord; few studies have focused on the mechanisms that regulate neuroinflammation and neuroprotection in the brain and spinal cord (9, 14, 17). We observed high expression levels of the immediate early gene *c-Fos* in SMC neurons after transcranial iTBS treatment, thus suggesting that the direct effect of transcranial iTBS was neuronal activation. These findings are consistent with the reported effects of TMS on neuronal activation (37). We speculated that the activation of neurons by transcranial iTBS reduced the number of nociceptive signals sent out by neurons, thus avoiding further recruitment and stimulation of microglia/macrophage activation. In addition, the reduced activation of microglia/macrophages further reduced damage to the neurons caused by the secretion of inflammatory factors (6, 35). The mechanism underlying these effects probably relates to the therapeutic effect of transcranial iTBS on the regulation of internal genes and the microenvironment of neurons. Therefore, the synergistic regulation of internal and external factors leads to a reduction in neuronal apoptosis.

After 4 weeks of transcranial iTBS, we performed transcriptomic sequencing of SMC tissue; the analysis of inflammation- and apoptosis-related mRNAs were similar to the immunofluorescence results obtained after two weeks of iTBS. The mRNA levels of these pro-inflammatory genes were up-regulated after SCI but were significantly down-regulated to almost normal levels after transcranial iTBS treatment. The mRNAs of anti-inflammatory genes were significantly down-regulated in the sham iTBS group and up-regulated in the iTBS group to almost normal levels. Thus, transcranial iTBS played an important role in regulating the balance of the inflammatory microenvironment in the SMC. In the sham iTBS group, we observed high mRNA levels of pro-apoptotic genes; however, the mRNA levels of anti-apoptotic genes were significantly lower. In the iTBS group, the expression levels of pro-apoptotic genes were significantly down-regulated; in contrast, the expression levels of anti-apoptotic genes were up-regulated to levels that were similar to normal rats. One possible reason for this is that iTBS activation of SMC neurons reduced the emission of nociceptive signals and, thus, reduced microglia/macrophage aggregation and inflammatory factor expression, thereby decreasing neuronal apoptosis (35).

To further confirm the mechanism involved, we performed interaction network analysis of genes related to neuronal activation and the regulation of inflammation and apoptosis in the SMC. Notably, *Pdpk1*, at the core regulatory position, was significantly expressed after transcranial iTBS treatment. *Pdpk1* is known to be regulated by c-Fos and regulates the protein kinase families A, G, and C by activating the phosphorylation of ribosomal protein S6 (38), thus affecting the survival and metabolism of neurons and participating in the regulation of homeostasis in the immune microenvironment. In addition, *Pdpk1* also plays an important role in neuronal survival, axonal regeneration, and synaptic plasticity by regulating protein kinase B, the mechanistic target of the rapamycin pathway (38). The regulation of individual genes, such as phosphatase and tensin homolog (*PTEN*), mechanistic target of rapamycin, or *Pdpk1*, can promote the regeneration of brain-derived nerve fibers to a certain extent after SCI (24, 38). However, our study suggested that maintaining homeostasis in the microenvironment was essential in promoting the regeneration of brain-derived nerve fibers at the site of injury while regulating the endogenous regeneration signal in SMC neurons.

### 4.2 Neuroprotective effects of transcranial iTBS on spinal cord

Inflammation, oxidative stress, and tissue necrosis at the site of SCI are far more serious than in the SMC. If the pathological processes at the site of injury are not controlled in time, the spinal cord neurons in the regions rostral and caudal to the injury site will continue to be lost (1). Understanding whether the effects of transcranial iTBS on the SMC can regulate the microenvironment at the site of injury and reduce inflammation and neuronal apoptosis as much as possible is crucial for promoting nerve regeneration and functional reconstruction; however, few studies have been published in this area (17, 39).

Our results suggested that transcranial iTBS could activate neurons in the spinal cord that receive neural information from the brain. After 4 weeks of transcranial iTBS treatment, inflammation and apoptosis analysis showed that the number of CD68^+^ microglia/macrophages and Cle-caspase3^+^ apoptotic neurons in the regions rostral and caudal to the injury site, as well as CD68^+^ microglia/macrophages at the injury site, were significantly lower in the iTBS group than in the Control and the sham iTBS group. This suggested that the activation of spinal cord neurons may be involved in regulating neuroinflammation to reduce apoptosis after receiving iTBS stimulation transmitted by brain-derived nerve fibers.

Transcriptomic sequencing of injured spinal cord tissue suggested that a large number of pro-inflammatory genes and pro-apoptotic genes were up-regulated after injury, while the expression levels of pro-inflammatory genes and pro-apoptotic genes were significantly reduced after transcranial iTBS treatment when compared with the sham iTBS group, although these levels were still higher than those in the Normal group. Further analysis of the interactions between genes related to neuronal activation and inflammation suggested that the downregulation of *TYRO protein tyrosine kinase-binding protein* after activation of spinal cord neurons played a key role in regulating complement genes *C1qa*, *C1qb*, and *C1qc* to maintain the stability of the immune microenvironment (40, 41). On the other hand, the down-regulated genes related to stimulus-response (*interferon regulatory factor 7*, *myxovirus resistance 1*, Major Histocompatibility Complex class II member *RT1-Ba*, *S100a8*, and *S100a9*) helped to avoid the hyperactivation of aberrant immune cells by transcranial iTBS. Therefore, this process plays an important role in regulating the balance of the immune microenvironment and avoiding the over-activation of inflammatory cells (42–44). In the sham iTBS group, we detected significant downregulation of genes related to neuronal activation, while genes related to microglia/macrophage activation, along with pro-inflammatory genes, were significantly up-regulated. Our gene ontology network diagram also suggested that transcranial iTBS activated spinal cord neurons to produce multidirectional regulatory effects on the immune microenvironment and also played an important regulatory role in neuronal survival and astrocyte activation.

Four weeks after SCI, the expression of *ATF3*, a marker of injured neurons, was also significantly higher in the rostral and caudal injury areas, thus suggesting that the progression of inflammation in the subacute phase was still causing damage to neurons at both ends of injury site (32). The expression of *ATF3* was significantly reduced in the iTBS group; this is consistent with the results of iTBS inhibiting neuroinflammation and reducing neuronal apoptosis, thus suggesting that transcranial iTBS played an important role in regulating homeostasis in the microenvironment, thus reducing neuronal injury and apoptosis. In addition, we also found that iTBS significantly reduced the expression of GFAP in the regions rostral and caudal to/in the injury site of the spinal cord. Transcriptomic sequencing also suggested that genes related to astrocyte activation were significantly up-regulated in the sham iTBS group when compared with the iTBS group. This is consistent with the effects of inflammatory factors on astrocyte activation and proliferation described in previous studies (45, 46). The inhibitory effect of transcranial iTBS on the excessive proliferation of astrocytes is beneficial as it can alleviate the obstacles of nerve fiber regeneration across the injury site; this is vital for the recovery of voluntary motor function. Furthermore, a scatter plot of key mRNAs showed that genes related to nerve regeneration were significantly up-regulated in the iTBS group, while genes that inhibited nerve regeneration were significantly up-regulated in the sham iTBS group.

### 4.3 The mechanism underlying the effects of transcranial iTBS on neuronal activation in the regulation of axonal regeneration and neuroplasticity

After confirming the neuroprotective effects of transcranial iTBS during the acute and subacute phases of SCI, we detected axonal regeneration at the injury site of the spinal cord. Analysis showed that the number of GAP43^+^ and NF^+^ nerve fibers in the regions rostral and caudal to/in the injury site in the iTBS group was significantly higher than in the Control group and sham iTBS group four weeks after SCI, thus confirming our hypothesis that transcranial iTBS could activate neurons in the brain and spinal cord and regulate the immune microenvironment to achieve neuroprotective effects and initiate axon regeneration. In addition to the significantly higher expression of GAP43 and NF, we also detected significantly higher expression of the synapse-associated proteins PSD95 and SYN, thus suggesting that transcranial iTBS not only promoted axonal regeneration but also affected synaptic plasticity (33).

Many studies have confirmed that regulating axon regeneration and neural pathway remodeling after nerve injury is an important prerequisite for the recovery of function (23, 24, 47, 48). We analyzed the interactions of genes related to neuronal activation, neurotransmitters, neuronal metabolism, nerve regeneration, and synaptic plasticity to investigate the key genes that regulate axonal regeneration and neural pathway remodeling after activation of spinal neurons by transcranial iTBS. Analysis showed that the down-regulation of *Pycard*, *Cd74*, and *Cyr61* regulated the expression of genes related to neurotransmitters and neural metabolism after the activation of spinal cord neurons by transcranial iTBS. Subsequently, transcranial iTBS regulated neuronal ion channels by upregulating *Scn5a* and regulated the neuronal cytoskeleton and axonal regeneration by up-regulating *Avil*. Transcranial iTBS also regulated neuronal dendritic remodeling and synaptic plasticity by down-regulating *Twist1*. Finally, we found that, after transcranial iTBS treatment, the joint motor ability of paralyzed hindlimbs was enhanced, and the injured spinal cord could transmit CMEPs with the highest peak amplitude. The results of behavioral tests fully confirmed the therapeutic action and regulatory effect of transcranial iTBS on axon regeneration and neuroplasticity in the injured spinal cord.

### 4.4 The neural pathway of transcranial iTBS that leads to the recovery of voluntary motor function

In order to further identify the neural pathways by which transcranial iTBS promotes the restoration of voluntary motor function after spinal cord transection in rats, we performed BDA tracing on the SMC and the LDPT of the cervical spinal cord after eight weeks of transcranial iTBS treatment. Analysis showed that BDA^+^ CST fibers regenerated more in the rostral region of injury after treatment, but it was difficult for these fibers to cross the injury site and regenerate the caudal region of injury. These data were not inconsistent with our previous observation that the numbers of GAP43^+^ and NF^+^ nerve fibers were significantly increased in the regions central and caudal to the injury site in the iTBS group.

Following injection into the SMC, BDA was detected in the raphe nucleus of the brain stem in the iTBS group; this suggested that after transcranial iTBS treatment, the motor neural circuit produced structural and functional remodeling, and that the CST might regenerate to the red and raphe nuclei in the midbrain (**Figures 9A, B**), in which the neurons could project to the spinal tracts (47) or to the neurons of the LDPT in the rostral injury region (**Figures 9C, D**), forming synapses with these neurons (**Figure S2**) (37). After receiving neuronal information *via* the CST, these neurons might regenerate and cross the injury area and eventually established synapses with the neurons that control hindlimb movement, ultimately mediating the restoration of voluntary motor function and electrophysiological function of the spinal cord (23, 24).

The cell bodies of 5-HT^+^ nerve fibers originate from the raphe nucleus of the midbrain. A large number of studies have confirmed that 5-HT^+^ nerve fibers have a stronger regenerative ability through injury sites than CST nerve fibers, and that this regeneration plays an important role in the repair of voluntary motor function (23–25). In particular, this process plays an important compensatory role in the limited regeneration of the CST (23). After transcranial iTBS, neurons in the raphe nucleus, which projects 5-HT nerve fibers, were highly activated and expressed c-Fos. Moreover, compared to the other groups, more 5-HT^+^ nerve fibers were observed to regenerate into the regions rostral and caudal to/in the injury site 8 weeks after SCI. The regeneration of the CST and 5-HT nerve fibers was positively correlated with the recovery of hindlimb motor function (47, 48).

Notably, c-Fos expression was detected in neurons activated by transcranial iTBS in the cervical spinal cord. The results of BDA tracing in the LDPT of the cervical spinal cord further suggested that there were more BDA^+^ nerve fibers in the regions rostral and caudal to/in the injury site in the iTBS group than the other groups. This data suggested that the neurons in the LDPT of the cervical spinal cord are most likely to regenerate across the injury site after receiving signals from CST or 5-HT nerve fibers and relay these to motor neurons in the caudal injury regions, thus further mediating the repair of motor function (**Figure 9H**).

## 5 Conclusion

In this study, we used a model of transected spinal cords in rats to reveal that transcranial iTBS treatment, commencing 72 hours after injury, could activate neurons in the brain and spinal cord to play a regulatory role in the homeostasis of the neuroimmune microenvironment of the brain and spinal cord. This could prevent neuronal injury and apoptosis while initiating axonal regeneration, ultimately restoring voluntary motor function by reconstructing motor pathways. Furthermore, our study revealed the key genes that regulate the neuroimmune microenvironment and initiate axonal regeneration and neuroplasticity after activating the neural pathway by transcranial iTBS. These genes could be used as regulatory targets to enhance therapeutic effects in subsequent studies. In addition, our study also highlighted new mechanisms underlying the effects of transcranial iTBS on the repair of severe SCI in the context of cells, proteins, and gene interactions. However, it should also be considered that it is difficult to achieve truly ideal motor function restoration with a single treatment for severe SCI. In addition to affirming the therapeutic value of transcranial iTBS, the value of transcranial iTBS combined with cutting-edge therapeutic strategies, such as stem cell transplantation, bioactive scaffolding, and tissue engineering therapy, should be considered to jointly solve the problems associated with recovering voluntary motor function and sensation after SCI.

## Data availability statement

The datasets presented in this study can be found in online repositories. The names of the repository/repositories and accession number(s) can be found below: PRJEB61293 (ENA).

## Ethics statement

The animal study was reviewed and approved by All animal protocols and animal handling procedures were approved by the ethics committee of Sun Yat-sen University (Animal Use Protocol no. 2021PS704K).

## Author contributions

B-QL, Y-SZ and L-XZ designed and supervised the study. J-LL, SW and Z-HC performed the experiments and collected the data. J-LL, SW, B-QL, Y-SZ and L-XZ summarized, analyzed, and plotted the data and drafted the manuscript. Z-HC, R-JW, H-YY, S-BY, JX, Y-NG, YD, GL, XZ, Y-HM, Y-LG and C-RW helped with study planning and critically reviewed the manuscript. J-LL, B-QL and SW wrote and finalized the article. All authors contributed to the article and approved the submitted version.
