# Supplementary material for: The therapeutic mechanism of transcranial iTBS on nerve regeneration and functional recovery in rats with complete spinal cord transection
Source: Front Immunol. 2023 Jun 14;14:1153516. doi: 10.3389/fimmu.2023.1153516 (PMC10306419; doi:10.3389/fimmu.2023.1153516)
Supplement: Supplementary file 1 [file DataSheet_1.pdf]

Supplementary Material

1 Supplementary Figures and Tables

1.1 Supplementary Figures

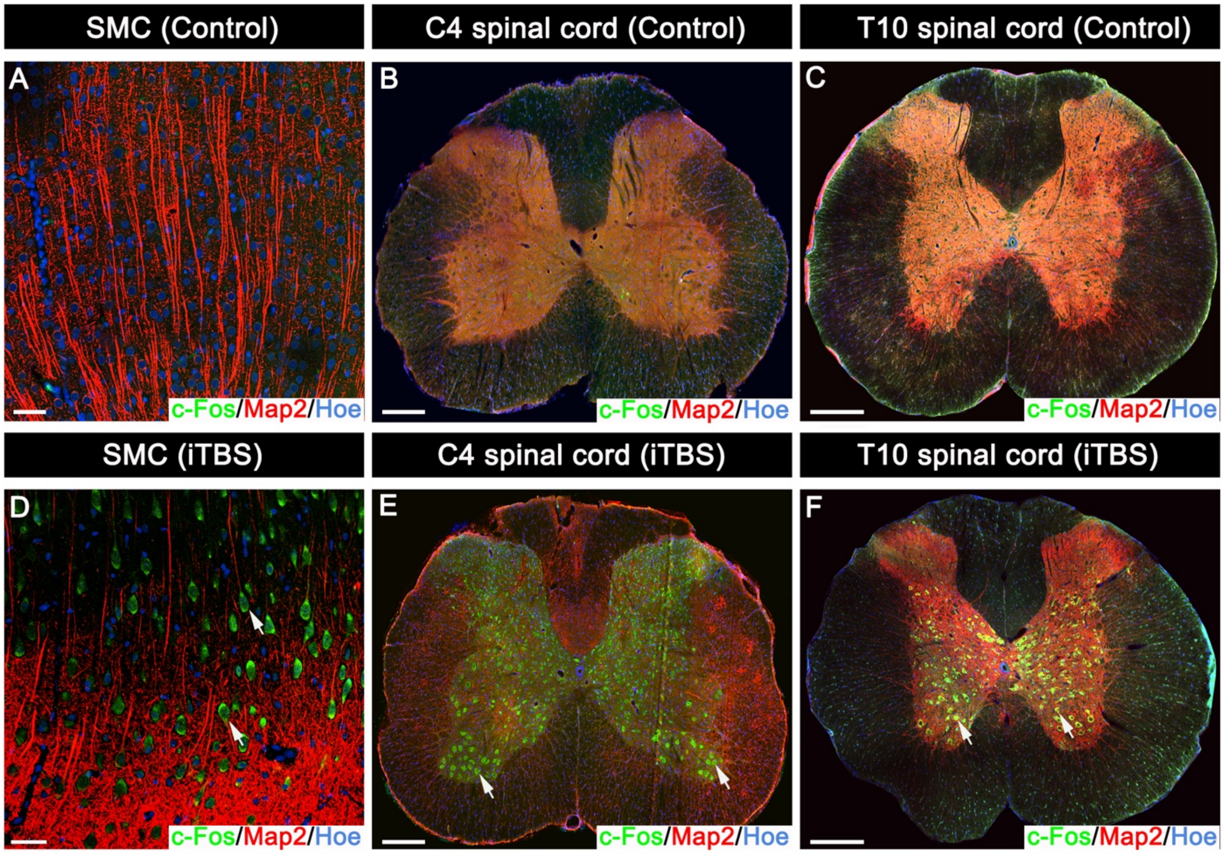

Supplementary Figure 1. Effects of transcranial iTBS on neuron activation. (A, D) The expression of c-Fos on the nucleus and perinuclear cytoplasm in $Map2^+$ neurons in the SMC of the Control group and the iTBS group in normal rats. (B, E) The expression of c-Fos in $Map2^+$ neurons at C4 level of spinal cord in the Control group and the iTBS group in normal rats. (C, F) The expression of c-Fos in $Map2^+$ neurons at T10 level of spinal cord in the Control group and the iTBS group in normal rats. Scale bars =50μm in (A) and (D); 300μm in (B), (C), (E) and (F).

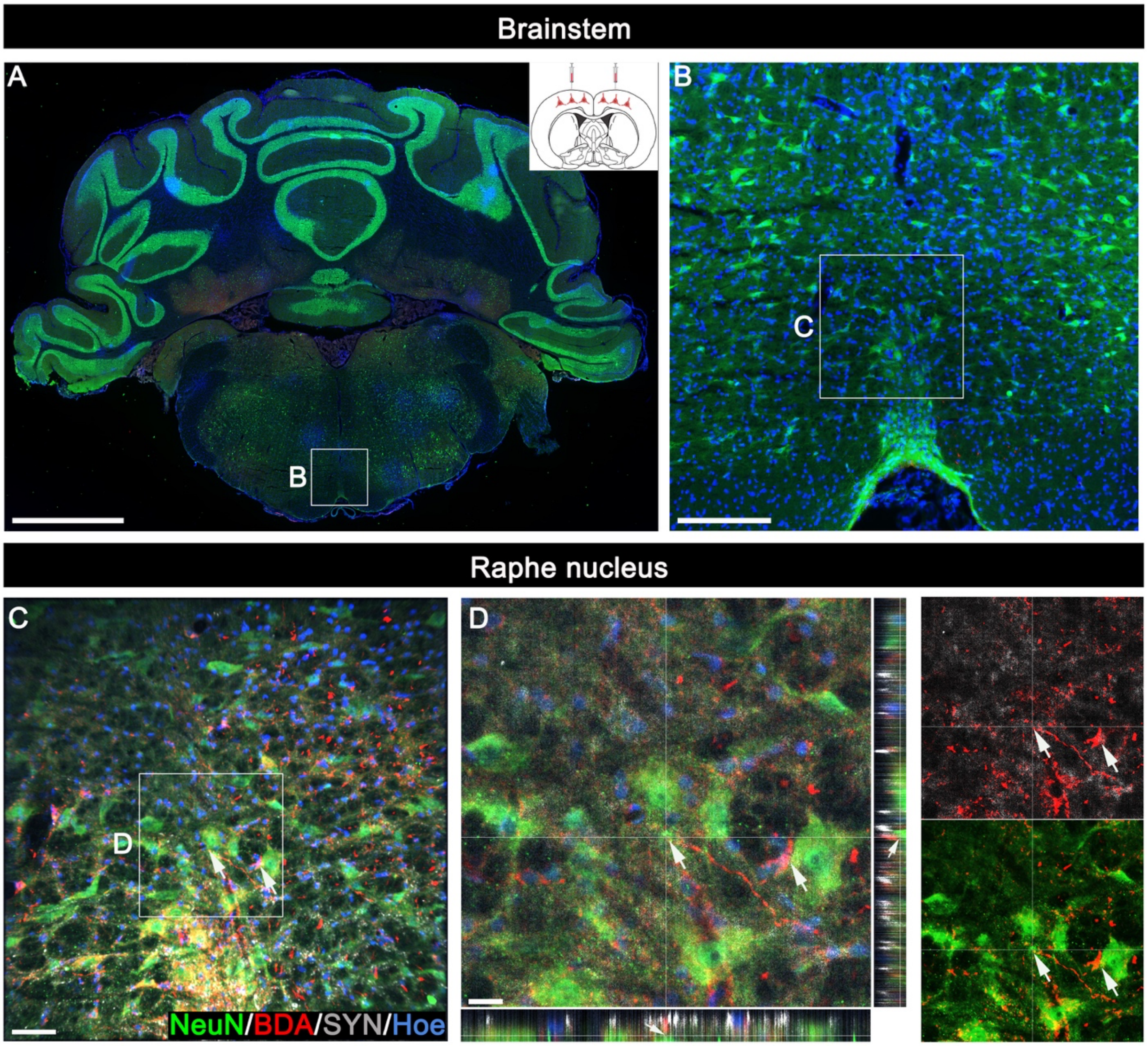

Supplementary Figure 2. Effects of transcranial iTBS on synaptic plasticity in the brainstem. (A) The co-expression of BDA and SYN in NeuN$^+$ neurons in the brainstem. (B) The picture is the magnification of the area indicated in the box area of (A), which showing the co-expression of BDA and SYN in NeuN$^+$ neurons in the raphe nucleus. (C) The picture is the magnification of the area indicated in the box area of (B), which showing the co-expression of BDA and SYN in NeuN$^+$ neurons in the raphe nucleus. (D) The picture is the magnification of the area indicated in the box area of (C), which showing the co-expression of BDA and SYN in NeuN$^+$ neurons in the raphe nucleus. Scale bars =2000μm in (A); 200μm in (B); 50μm in (C); 20μm in (D).

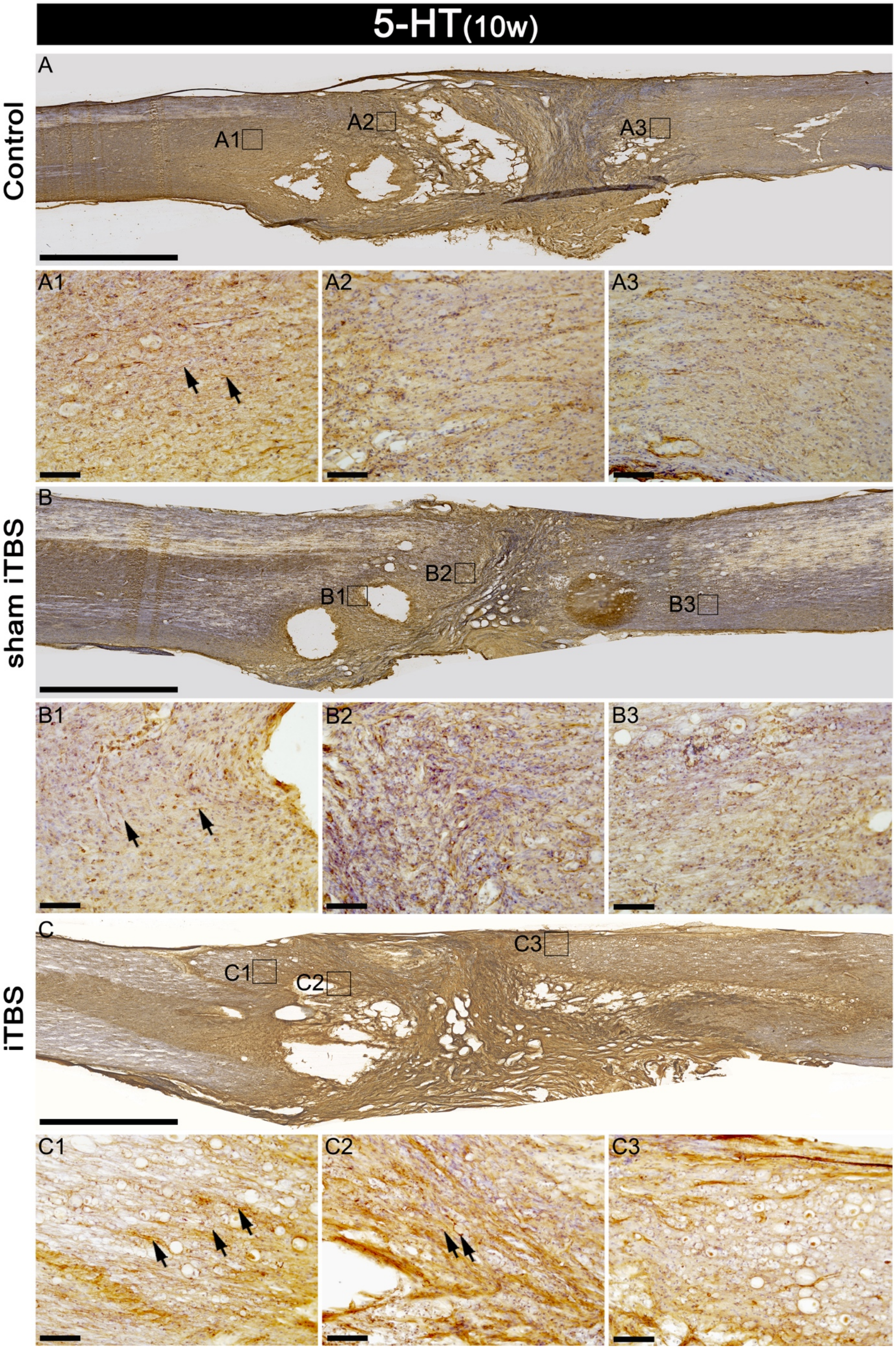
5-HT(10w)
Control
A
A1
A2
A3
sham iTBS
B
B1
B2
B3
iTBS
C
C1
C2
C3

Supplementary Figure 3. Effects of transcranial iTBS on regeneration of 5-HT positive nerve fibers. (A-C) Low magnification of sagittal spinal cord section showing the expression of 5-HT. (A1, -C3) The pictures are the magnification of the area indicated in the box area of (A-C). Arrows indicate 5-HT$^+$ nerve fibers. Scale bars =2000μm in (A) - (C); 40μm in (A1) - (C3).

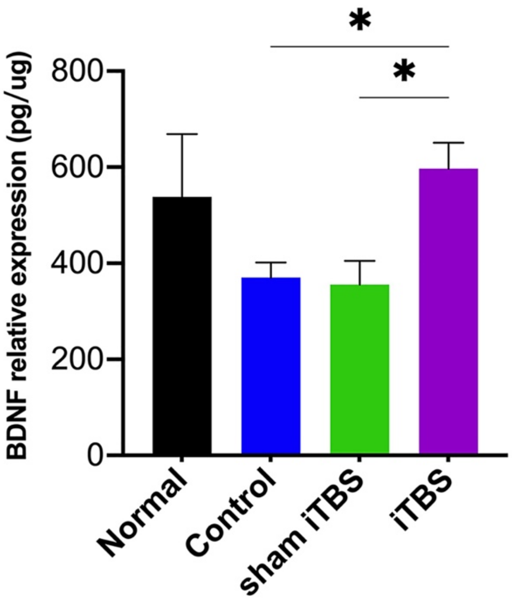

Supplementary Figure 4. Effects of transcranial iTBS on expression of BDNF in the rostral region to the injury site of spinal cord at weeks 4. Bar chart showing the relative expression of BDNF measured by ELISA in the rostral region to the injury site of spinal cord (*$P < 0.05$). Data are presented as mean ± SEM ($n = 5$).

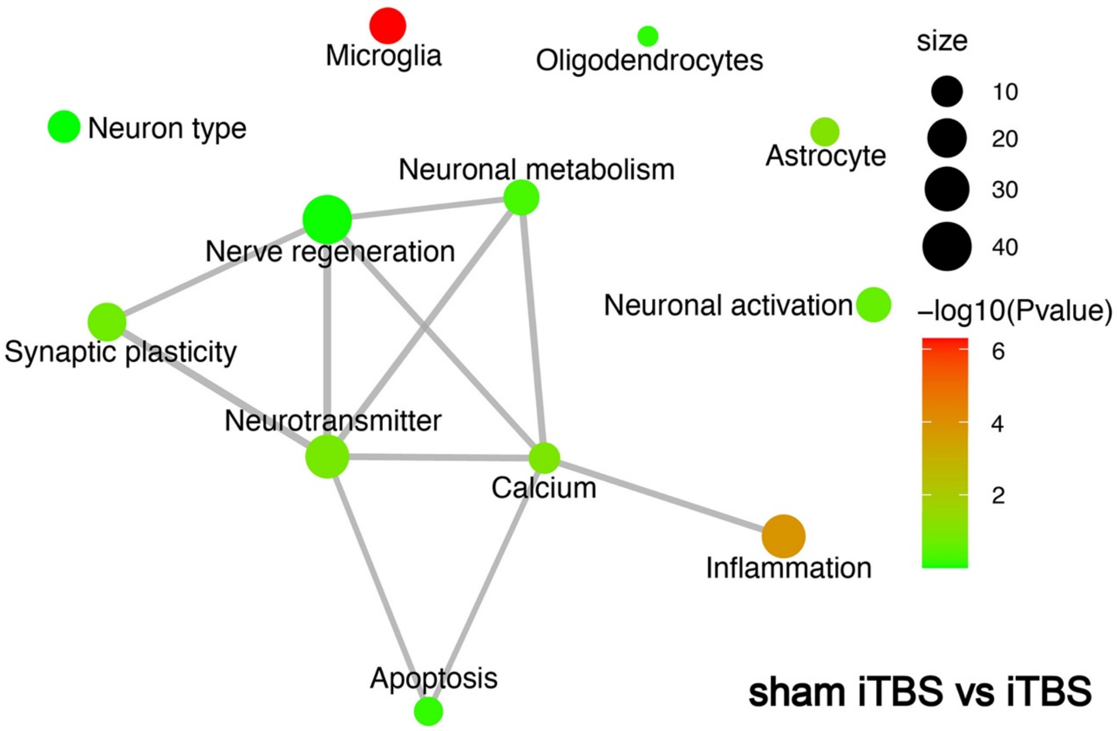

Supplementary Figure 5. The network diagram showing the relationship between the designated signal pathways and the enriched genes. Each node is displayed in color according to -Log10 (*P*-value), and the node size is displayed according to the number of genes.

1.2 Supplementary Tables

Supplementary Table 1: Tissue preparation processes

Time point	Sampling point	Tissue preparation
2w (*n* = 5 in each group)	SMC- IF	5 rats in each group were perfused with PBS as described previously. Whole brains were removed by craniotomy. One side of the brain hemisphere was fixed with 4% paraformaldehyde (PFA) at 4 ℃ for 3 d, followed by dehydration with 30% sucrose solution for 2 weeks.
	SMC- WB	The SMC tissue with a length of 5mm of the other side (2 mm off the coronal suture and 3 mm off the sagittal suture) was removed carefully and washed in PBS. Next, put the tissue directly into a tube containing the configured RIPA Lysis Buffer Eppendorf. Then, the tissue was broken up by ultrasound and was placed on ice for 20 min and centrifuged at 12000 rpm for 20 min at 4 ℃. Finally, the protein supernatant was extracted and placed at -80℃.
4w (*n* = 5 in each group)	SMC- mRNA sequencing	5 rats in each group were perfused with PBS as described previously. The SMC tissue with a length of 5mm was removed carefully and washed in diethylpyrocarbonate-treated ddH2O and immediately put into liquid nitrogen for quick freezing. Frozen tissues were immediately stored at - 80 ℃. RNA extraction was then performed.
	C4 SC- IF	The spinal cord tissue sections with a length of 2-cm were removed with C4 as the center and fixed in 4% PFA at 4 ℃ for 3 d, followed by dehydration

	with 30% sucrose solution for 2 weeks.
T10 SC- mRNA sequencing	The spinal cord tissue sections with a length of 5mm in the rostral injury region of T10 were removed on ice and divided into two parts. One part was washed in diethylpyrocarbonate-treated ddH2O and immediately put into liquid nitrogen for quick freezing. Frozen tissues were immediately stored at -80 °C. RNA extraction was then performed.
T10 SC- IF	The spinal cord tissue sections with a length of 2-cm were removed with T10 as the center and fixed in 4% PFA at 4 °C for 3 d, followed by dehydration with 30% sucrose solution for 2 weeks.
T10 SC- WB	The spinal cord tissue sections with a length of 5mm in the rostral injury region of T10 were removed on ice and divided into two parts. The other part of the tissue was directly into a tube containing the configured RIPA Lysis Buffer Eppendorf. Then, the tissue was broken up by ultrasound and was placed on ice for 20 min and centrifuged at 12000 rpm for 20 min at 4 °C. Finally, the protein supernatant was extracted and placed at -80°C.
T10 SC- ELISA	The spinal cord tissue sections with a length of 5mm in the rostral injury region of T10 were removed on ice and divided into two parts. The other part of the tissue was directly into a tube containing the configured RIPA Lysis Buffer Eppendorf. Then, the tissue was broken up by ultrasound and was placed on ice for 20 min and centrifuged at 12000 rpm for 20 min at 4 °C. Finally, the protein

		supernatant was extracted and placed at -80℃.
10w (*n* = 5 in the Control group, the sham iTBS group and the iTBS group)	Raphe nucleus- IF	5 rats in the Control group, sham iTBS group, and iTBS group were perfused were perfused with PBS as described previously and with 4% PFA until shaking their limbs and wagging their tails. The total perfusion amount of 4% PFA in each rat was about 100 ml. Then, brain stem tissue sections were removed and fixed with 4% PFA at 4 ℃ for 2 d, followed by dehydration with 30% sucrose solution for 2 weeks.
	C3 SC- IF	Same as above for the animal perfusion. Then, the spinal cord tissue sections with a length of 2-cm were removed with C3 as the center and fixed with 4% PFA at 4 ℃ for 2 d, followed by dehydration with 30% sucrose solution for 2 weeks.
	T10 SC- IF	Same as above for the animal perfusion. Then, the spinal cord tissue sections with a length of 2-cm were removed with T10 as the center and fixed with 4% PFA at 4 ℃ for 2 d, followed by dehydration with 30% sucrose solution for 2 weeks.
	T10 SC- DAB	Same as above for the animal perfusion. Then, the spinal cord tissue sections with a length of 2-cm were removed with T10 as the center and fixed with 4% PFA at 4 ℃ for 2 d, followed by dehydration with 30% sucrose solution for 2 weeks.

Abbreviation: iTBS (intermittent theta-burst stimulation); SMC (sensorimotor cortex); IF (immunofluorescence); WB (western Blot); SC (spinal cord); ELISA (enzyme-linked immunosorbent assay); DAB (3,3'-diaminobenzidine).

Supplementary Table 2. List of antibodies.

Antibodies	Tedia Company Inc	Dilution Rate
IBA-1 (Rabbit)	Wako（SKN4887, Japan）	1:250（IF）
CD206 (Rabbit)	Abcam（ab64693, UK）	1:1000（WB）
CCR7 (Rabbit)	Abcam（ab32527, UK）	1:1000（WB）
CD68 (Rabbit)	Cell signaling Technology (E307V, US)	1:250（IF）
Pro-caspase3 (Rabbit)	Beyotime（AF6432, China）<br>Cell signaling Technology (9662S, US)	1:1000（WB）<br>1:1000（WB）
Cleaved-caspase3 (Rabbit)	Cell signaling Technology (D175, US)	1:1000（WB）<br>1:250（IF）
GAPDH (Rabbit)	Sigma (SAB4300645, US)	1:2000（WB）
β-actin (Rabbit)	Proteintech (00109130, US)	1:2000（WB）
ATF3 (Rabbit)	Sigma (HPA001562, US)	1:250（IF）
GFAP (Mouse)	Abcam（ab4648, UK）	1:250（IF）
GAP43 (Rabbit)	Sigma (SAB4300525, US)	1:250（IF）
NeuN (Mouse)	Abcam（ab104224, UK）	1:300（IF）
(Rabbit)	Abcam（ab104225, UK）	1:300（IF）
NF (Mouse)	Biolegend (B333596, US)	1:300（IF）
PSD95 (Rabbit)	Abcam（ab18285, UK）	1:1000（WB）
SYN (Mouse)	Abcam（ab8049, UK）	1:500（WB）
NF200 (Rabbit)	Sigma (N4142, US)	1:250（IF）<br>1:1000（WB）

MAP2 (Rabbit)	Sigma (M3696, US)	1:500（IF）
5-HT (Rabbit)	Sigma (S5545, US)	1:1000 (DAB)
c-Fos (Mouse)	Santa Cruz (sc-8047, US)	1:50（IF）
HRP (Rabbit)	Abcam（ab205718, US）	1:2000（WB）
HRP (Mouse)	Abcam（ab6789, US）	1:2000（WB）
Alexa FluorⓇ488	Abcam（ab150077, US）	1:600（IF）
Alexa FluorⓇ555	Abcam（ab150169, US）	1:600（IF）
Alexa FluorⓂ488	Abcam（ab150113, US）	1:600（IF）
Alexa FluorⓂ555	Abcam（ab150114, US）	1:600（IF）
Alexa FluorⓂ647	Abcam（ab150115, US）	1:600（IF）
Streptavidin Conjugate555	Invitrogen (S32355, US)	1:300（IF）
Hoechst33342	Beyotime (C1022, China)	1:500（IF）

Supplementary Table 3. List of the Log2FC value on different genes of different brain tissue samples.

	**Normal**	**Normal**	**sham iTBS**	**sham iTBS**	**sham iTBS**	**iTBS**	**iTBS**	**iTBS**
Rbp4	8.50	7.92	9.23	9.84	9.46	8.66	8.86	7.78
Sp110	6.40	6.07	7.87	7.78	7.97	7.08	7.08	7.04
Cd37	5.78	5.73	6.96	7.23	7.80	5.93	6.62	6.13
Rps19	9.99	10.77	11.44	11.41	12.36	11.30	10.87	10.41
Trem2	7.62	8.05	8.74	8.67	9.55	8.46	8.06	7.94
Crlf2	6.53	6.79	7.51	7.84	8.22	7.19	7.31	6.75
Tyrobp	7.78	7.66	8.92	9.24	9.84	8.56	8.26	7.85

C1qa	10.35	10.68	11.54	11.87	12.12	10.99	11.17	10.25
Bloc1s3	5.20	5.41	6.42	6.80	7.12	6.05	6.12	5.24
Tnfaip8l2	7.62	7.28	8.25	8.42	9.04	7.78	8.04	7.06
C1qc	9.32	9.64	10.57	10.68	11.17	9.65	10.37	9.44
RT1-DMa	8.03	8.27	9.11	9.03	9.69	8.28	8.86	8.02
C1qb	9.98	10.03	11.01	11.38	11.48	10.19	10.86	9.70
S100a8	0.00	2.18	3.19	3.46	4.71	2.11	2.21	0.00
Fcer1g	8.98	8.88	9.90	9.97	10.42	9.33	9.61	8.88
Tmem176a	9.61	9.60	10.44	10.60	11.28	10.40	10.42	9.58
Ccl24	4.07	3.39	4.85	5.04	5.71	4.60	4.18	3.95
RT1-Ba	4.95	3.68	5.56	6.60	7.16	4.39	4.81	3.84
Pycard	5.47	5.45	5.76	6.35	6.82	5.76	5.41	4.54
Itga2b	5.13	5.09	6.16	6.96	6.67	5.95	6.12	4.90
Gpsm3	6.73	6.28	7.44	8.10	8.25	7.31	6.91	5.74
Sectm1a	3.92	4.55	5.33	5.94	6.41	5.31	4.50	5.32
Oasl	3.84	0.00	4.00	5.26	5.23	0.00	3.04	2.12
Cxcl9	0.00	0.00	3.36	3.46	4.12	0.00	0.00	0.00
Rtp4	6.06	5.41	6.92	8.00	7.41	6.03	6.16	6.15
Irf7	5.99	5.23	6.40	8.00	7.30	6.19	5.92	6.06
S100a9	1.41	2.52	3.19	4.87	3.89	0.00	0.00	2.12
Tap1	5.47	6.22	6.42	7.07	7.23	6.34	6.23	6.36
Mx1	4.99	4.82	6.60	7.30	7.68	6.10	6.28	6.48
Gzmm	6.66	7.12	7.94	7.92	8.75	7.70	7.80	7.26
Lat2	5.26	5.19	5.91	6.16	5.78	5.54	5.69	5.61
Cxcl17	4.74	4.23	5.45	5.80	5.88	2.71	4.90	4.54
Cd6	5.78	6.17	7.29	6.97	7.61	6.10	6.86	6.81
Gpr171	2.94	4.76	0.00	1.90	2.25	3.30	2.46	3.46
Samsn1	4.14	3.92	0.00	1.90	0.00	3.95	4.11	3.31
Il1r1	7.46	6.41	5.38	4.63	3.62	5.54	5.44	6.90
Itgb3	4.14	2.52	2.16	0.00	0.00	2.44	3.19	3.46
Ccr5	9.08	8.81	8.18	7.98	7.00	8.53	9.36	9.31
Angpt1	5.30	4.88	4.10	3.78	0.00	5.31	5.83	6.56
Peli2	7.79	7.64	7.26	6.85	4.90	7.38	7.83	8.90
Yes1	6.98	6.95	6.14	5.80	3.29	6.78	7.34	7.83
Atp11c	6.54	5.97	5.45	4.40	0.00	5.93	6.64	7.31
Zbtb7a	10.72	10.39	9.88	9.71	8.74	10.12	10.09	10.64
Dlg1	12.49	12.38	11.96	11.81	10.63	12.09	12.33	12.55

Rcor1	7.76	7.36	6.55	6.32	3.90	6.60	7.17	7.45
Cplx2	14.57	14.77	14.13	13.62	12.19	14.26	14.56	15.31
Plcb1	12.38	11.60	10.93	11.04	9.83	11.21	11.70	12.01
Inhba	6.71	5.32	3.19	3.46	0.00	4.90	5.32	6.18
Foxo3	9.73	8.83	7.09	7.30	5.32	7.84	8.26	9.56
Cacnb4	10.95	10.07	8.65	8.34	6.13	9.04	9.79	11.16
Adam10	10.96	10.34	9.98	9.83	8.75	10.23	10.53	11.06
Tsc1	9.90	9.39	8.62	8.09	6.11	9.15	9.29	10.56
Lgr4	9.85	9.61	8.77	8.70	7.01	9.18	9.57	10.33
Camk1d	11.37	10.80	10.18	9.67	8.15	10.33	10.84	11.41
Kif5b	12.28	12.30	11.55	10.98	9.77	11.99	12.29	13.40
Gab2	8.89	8.53	8.15	7.85	6.08	8.67	8.74	9.75
Bmi1	9.34	8.78	8.18	8.06	6.74	8.79	8.96	9.59
Gpr17	9.01	8.36	7.91	7.28	6.30	7.96	8.83	9.59
Zbtb16	9.44	8.30	7.05	5.65	5.53	8.08	7.82	9.53
Ppargc1b	6.66	5.19	3.78	2.48	3.09	4.90	6.12	7.15
Cadm1	10.87	9.82	9.18	9.09	8.35	9.34	10.64	11.19
Cbl	8.14	6.24	5.06	4.27	3.47	5.54	6.90	8.96
Asxl2	7.88	5.94	5.88	5.73	3.89	6.00	7.22	8.62
Wdfy1	8.34	7.63	6.36	6.35	4.84	6.97	7.92	8.92
Rbfox2	10.01	9.05	8.31	7.84	6.37	8.37	9.32	10.37
Traf6	7.11	6.33	4.96	5.26	3.62	5.60	6.25	7.62
Braf	8.76	8.25	7.75	7.60	6.65	7.77	8.33	9.29
Zbtb46	7.65	7.06	6.07	5.73	5.23	6.41	7.24	7.59
Satb1	11.58	11.39	10.25	9.77	8.78	10.71	10.44	11.95
Hrh2	6.35	6.71	5.11	4.33	2.86	5.64	5.79	7.12
Prkcb	12.91	12.36	11.30	11.28	10.68	11.77	11.77	13.00
Klf13	7.75	6.85	6.40	5.00	4.22	6.56	7.19	8.38
Traf3	11.59	11.52	10.85	9.93	8.88	10.86	11.12	12.27
Tmf1	10.80	10.28	9.68	9.32	8.56	9.73	10.11	10.92
Pdpk1	9.67	8.33	6.92	6.44	4.71	7.74	8.69	10.28
Bmpr1a	10.30	9.78	9.26	8.70	7.49	9.43	10.01	10.75
Wnt1	4.14	3.68	2.16	2.22	0.00	4.31	3.95	5.06
Serpinc1	4.33	4.32	2.16	2.48	0.00	3.84	3.95	4.96
Styk1	4.07	3.02	2.16	1.90	0.00	2.44	4.11	2.72
Pik3r1	11.77	11.15	10.52	10.40	9.98	10.62	10.58	11.25

Prdm1	4.74	4.55	2.76	2.48	2.86	4.31	4.03	4.32
Lingo1	13.62	13.26	13.17	13.18	13.38	12.88	13.06	12.94
Glp1r	0.00	0.00	3.19	3.96	2.25	2.11	0.00	0.00
Traf1	3.92	3.68	4.20	4.92	5.18	2.71	3.67	3.46
Mdk	8.97	9.30	10.03	9.94	10.96	9.79	9.68	9.14
Bcl2a1	4.87	5.14	6.32	6.23	6.74	5.73	5.18	5.61
Ifi27	7.90	9.40	9.49	10.23	10.23	9.15	9.05	8.61
Fnip2	8.71	7.98	7.25	6.97	4.12	7.44	7.99	9.12
Ppargc1a	9.31	8.25	6.65	6.94	4.71	6.99	8.19	9.85
Deptor	8.03	7.15	6.34	6.04	4.96	6.61	6.97	7.81
Neo1	10.90	10.11	9.87	9.54	8.67	10.11	10.40	11.20
Abcc1	8.45	7.91	7.22	6.89	5.49	7.42	8.23	9.49
Xiap	8.48	7.07	6.01	5.78	3.29	6.32	7.70	9.52
Birc6	10.21	9.16	7.29	7.43	5.41	8.24	9.43	11.08
Dnajc5	10.73	9.88	8.94	8.98	7.94	9.29	10.23	10.50
Gabrb2	9.85	8.28	6.34	5.96	4.22	6.70	8.32	9.65
Dnm1l	11.88	11.37	10.55	10.51	9.27	10.76	11.41	11.77
Acvr1c	7.76	6.85	4.59	5.43	3.76	5.47	6.09	6.94
Eya4	6.57	5.45	4.73	4.05	0.00	5.27	4.90	6.72
Pten	11.17	10.55	9.89	9.73	9.15	10.13	10.31	11.33
Hipk1	11.86	11.62	11.20	10.51	9.96	11.30	11.43	12.57
Gsk3b	10.05	8.73	7.85	7.33	7.56	8.23	8.97	10.55
Dcc	7.95	6.85	5.45	5.33	5.13	5.67	7.27	8.43
Top2a	6.61	4.99	5.82	3.46	5.57	6.34	6.05	6.26

Supplementary Table 4. List of the Log2FC value on different genes of different spinal cord tissue samples.

	**Normal**	**Normal**	**Normal**	**sham iTBS**	**sham iTBS**	**sham iTBS**	**iTBS**	**iTBS**	**iTBS**
Rbfox2	8.06	8.09	7.80	7.56	8.33	8.22	9.06	8.42	8.49
Atp11c	7.31	7.16	6.72	7.03	7.72	7.75	8.31	7.81	7.89
Hrh2	5.59	5.20	5.66	5.08	4.98	5.31	6.24	5.48	5.43
Gpr17	9.70	9.26	9.14	8.60	9.36	9.32	9.79	9.37	9.46
Bmi1	9.54	9.18	9.36	9.49	9.70	9.70	9.91	9.74	9.79
Lig4	9.17	8.83	8.70	8.59	9.01	9.01	9.43	9.09	9.26
Rb1	10.02	9.76	9.43	10.06	10.18	10.12	10.57	10.20	10.60

Cadm1	9.74	9.46	9.87	9.75	10.08	10.12	10.45	10.21	10.85
Sectm1a	5.65	4.70	4.39	5.62	6.10	5.68	5.42	5.54	5.20
Gpsm3	7.17	7.69	7.11	8.17	8.75	8.57	8.04	7.88	8.13
Ifit3	5.77	6.30	4.88	8.09	9.11	8.57	8.02	5.96	7.75
Sp110	8.32	8.29	8.01	8.99	9.38	9.26	8.89	8.38	8.76
Aif1	9.18	9.07	8.48	10.22	10.41	10.28	9.74	9.98	9.62
Fcgr3a	7.45	7.19	6.61	8.54	9.14	8.89	8.07	8.15	7.89
Tyrobp	8.47	8.87	8.73	10.55	11.31	11.10	10.25	10.16	9.86
Rac2	8.41	8.20	8.12	9.18	10.02	9.77	9.16	8.82	8.99
Tagap	6.29	5.95	6.28	7.67	7.94	7.92	7.03	6.92	7.23
Tnfsf13b	5.74	5.43	4.07	7.36	7.58	7.88	7.05	6.26	6.99
Ikzf3	0.00	0.00	1.64	3.41	2.87	3.28	1.73	2.85	2.25
RT1-DMa	8.26	8.29	8.35	9.04	9.18	9.07	8.40	8.62	8.65
C1qb	10.50	10.38	10.44	12.20	12.84	12.55	11.21	11.38	11.31
C1qa	11.24	11.06	10.94	12.90	13.61	13.11	11.94	11.97	11.67
C1qc	9.93	9.12	9.61	11.09	11.93	11.35	10.16	10.20	9.94
Tnfaip8l2	7.57	7.68	7.60	9.01	9.52	9.42	8.34	8.61	8.66
Trem2	7.73	7.97	7.35	9.62	10.57	10.15	9.20	9.34	9.08
Cd37	6.96	6.95	6.60	8.27	8.79	8.52	8.06	7.66	7.83
Fcer1g	9.71	9.34	9.33	11.15	11.95	11.81	11.03	10.83	10.99
Cyba	7.90	7.39	7.27	9.10	9.65	9.66	8.93	8.54	8.72
Tmem176a	10.69	10.20	10.19	11.54	12.11	12.05	11.45	11.27	11.31
Abcc1	9.02	7.78	7.98	8.35	9.04	9.09	9.53	9.22	9.22
Snrk	9.87	9.61	9.80	9.35	9.61	9.76	9.88	9.90	9.85
Dnajc5	9.64	9.37	9.67	9.27	9.93	9.71	10.08	9.96	9.95
Hdac4	8.74	7.86	8.58	8.23	8.65	8.78	9.44	8.80	8.85
Eya4	7.97	6.81	7.00	6.53	7.16	6.96	7.52	7.22	7.72
Brsk2	10.14	9.92	9.93	9.84	9.78	9.57	10.01	10.13	9.98
Bcl2a1	6.50	6.49	6.07	8.17	9.03	8.29	7.36	7.74	7.60
Mdk	9.27	9.70	9.80	10.09	9.89	9.97	9.73	9.83	9.55
Relb	7.28	6.30	6.55	7.47	7.70	7.44	6.95	7.39	6.87
Nfkb1	9.60	9.37	9.62	10.25	10.48	10.35	10.22	9.91	10.05
Gusb	8.65	8.68	8.54	9.30	9.54	9.43	9.09	8.79	8.92
Ska1	3.85	4.28	4.39	4.27	4.81	4.60	2.17	3.96	2.86
Hopx	11.10	11.06	11.02	11.33	11.26	11.25	10.82	11.10	10.41
Ninj1	9.02	9.26	9.21	9.52	9.21	9.23	8.79	9.05	8.90

Cryab	12.13	12.55	12.82	12.35	12.11	11.98	11.37	11.95	11.97
Onecut2	2.12	0.00	0.00	2.28	1.48	2.96	0.00	0.00	0.00
Apoe	16.05	16.19	16.11	17.19	17.19	17.62	16.73	16.89	16.96
Serpinb1a	8.40	8.52	8.55	8.99	9.02	9.22	8.36	8.48	8.67
Ier2	7.55	7.50	7.47	7.96	8.18	8.21	7.73	7.53	7.85
Tox2	8.63	8.69	9.14	8.79	8.82	8.82	8.61	9.19	9.00
Glra2	7.77	7.46	7.98	7.03	7.36	7.56	7.51	7.83	7.96
Syt6	9.50	9.31	9.45	9.32	8.99	9.35	9.31	9.50	9.99
Vash2	7.92	7.56	7.84	7.55	7.25	7.41	7.57	7.41	7.51
Vwc2	8.15	7.68	7.93	7.68	7.13	6.99	7.38	7.68	7.71
Tnfaip8l3	7.04	6.24	6.66	7.39	8.31	7.37	7.35	7.04	6.67
Cldn9	5.55	5.76	4.66	6.34	6.36	6.20	5.02	5.67	5.57
Anxa4	10.35	10.15	10.23	11.04	11.29	11.18	10.92	10.45	10.96
Rspo1	3.85	4.87	4.83	8.03	7.71	8.03	7.48	7.24	7.08
Cxcl1	0	0	0	2.87	4.54	0	0	1.53	0
S100a8	9.25	5.56	7.02	8.68	9.10	4.80	4.44	0	3.86
S100a9	10.19	6.82	8.53	9.98	10.15	6.24	5.37	0	5.73
Ccl21	0	0	0	0	0	0	0	2.05	2.17
Wnt1	1.06	0	0	0	0	0	2.63	0	2.50
Lst1	3.91	0	2.45	5.28	5.86	5.42	3.37	4.01	4.20
Col3a1	8.91	7.40	11.05	7.42	11.31	12.77	7.32	7.71	11.04
Scn5a	4.97	4.46	6.02	4.55	5.16	4.76	6.12	5.73	5.89
Slc22a6	2.68	0	7.12	1.89	6.34	5.71	0	3.20	4.60
Cyp26c1	0	0	2.72	1.89	0	0	2.63	1.53	3.00
Nrgn	9.51	9.72	9.66	8.04	9.21	8.48	8.67	10.01	9.86
Cd74	9.99	8.36	11.17	12.24	12.83	12.35	11.28	11.75	11.58
Tyrobp	8.87	8.73	8.47	11.31	11.10	10.55	10.16	9.86	10.25
Ptger3	1.66	2.06	2.72	6.81	6.00	4.95	5.30	3.89	3.00
Irf7	7.65	7.33	8.41	10.23	9.80	9.72	8.18	8.94	8.93
Mx1	7.68	6.79	8.18	10.90	10.32	10.06	9.05	9.13	9.34
RT1-Ba	7.40	4.53	8.47	9.49	9.90	8.92	8.29	8.19	8.06
Cyr61	6.11	5.39	5.74	6.68	6.52	6.52	5.44	5.73	6.24
Cyp1b1	4.81	3.88	7.03	4.81	7.21	8.42	4.69	4.48	6.47
Avil	6.22	6.63	7.08	5.21	5.86	6.96	6.77	6.35	7.92
Twist1	5.57	2.87	5.34	4.44	5.47	6.19	3.96	3.09	4.41
Pycard	6.56	5.03	6.50	8.06	7.41	6.85	6.82	6.34	6.66
Cxcl17	4.28	1.64	4.32	6.31	6.39	6.24	4.63	5.25	5.88

F2rl1	4.20	2.06	4.15	6.15	6.16	5.94	4.80	4.60	5.34
